# Diverse maturity-dependent and complementary anti-apoptotic brakes safeguard human iPSC-derived neurons from cell death

**DOI:** 10.1038/s41419-022-05340-4

**Published:** 2022-10-21

**Authors:** Ruven Wilkens, Anne Hoffrichter, Karolina Kleinsimlinghaus, Bettina Bohl, Carolin Haag, Nadja Lehmann, Malin Schmidt, Elena Muñoz Perez-Vico, Julia Wangemann, Klara Franziska Rehder, Sandra Horschitz, Georg Köhr, Julia Ladewig, Philipp Koch

**Affiliations:** 1 Department of Translational Brain Research, Central Institute of Mental Health (ZI), University of Heidelberg/Medical Faculty Mannheim, Mannheim, Germany; 2 HITBR Hector Institute for Translational Brain Research gGmbH, Mannheim, Germany; 3 German Cancer Research Center (DKFZ), Heidelberg, Germany; 4 Department of Neurophysiology, Medical Faculty Mannheim, University of Heidelberg, Mannheim, Germany

**Keywords:** Differentiation, Cell death in the nervous system, Pluripotent stem cells

## Abstract

In humans, most neurons are born during embryonic development and have to persist throughout the entire lifespan of an individual. Thus, human neurons have to develop elaborate survival strategies to protect against accidental cell death. We set out to decipher the developmental adaptations resulting in neuronal resilience. We demonstrate that, during the time course of maturation, human neurons install a complex and complementary anti-apoptotic signaling network. This includes i.) a downregulation of central proteins of the intrinsic apoptosis pathway including several caspases, ii.) a shift in the ratio of pro- and anti-apoptotic BCL-2 family proteins, and iii.) an elaborate regulatory network resulting in upregulation of the inhibitor of apoptosis protein (IAP) XIAP. Together, these adaptations strongly increase the threshold for apoptosis initiation when confronted with a wide range of cellular stressors. Our results highlight how human neurons are endowed with complex and redundant preemptive strategies to protect against stress and cell death.

## Introduction

Neurons represent a highly specialized cell population of the central and peripheral nervous system responsible for communicating information throughout the body. Since replacement of larger numbers of neurons is virtually impossible for the adult human organism, their protection is of utmost importance to maintain systemic integrity.

Apoptotic cell death is regulated via an extrinsic and an intrinsic signaling pathway. The extrinsic pathway relies on the activation of transmembrane death receptors by immune cells or inflammatory cytokines. In contrast, the intrinsic pathway for programmed cell death is initiated upon disruption of cellular homeostasis which - in the brain - is in most cases caused by hypoxia, oxidative damage or aberrant protein folding. These events cause oligomerization of the BCL-2 family proteins, disruption of mitochondrial membrane integrity and leakage of Cytochrome c into the cytosol [1]. Cytochrome c and the cytosolic protein apoptotic protease activating factor 1 (APAF-1) form the apoptosome which serves as a scaffold for the activation of initiator Caspase-9 and subsequently effector Caspases-3 and -7. The latter eventually trigger events of the execution phase of apoptosis. Signaling events along this apoptotic cascade may be counteracted by a variety of pro-survival factors, most prominently members of the BCL-2 family and inhibitor of apoptosis proteins (IAPs) [2–4]. During development, programmed cell death plays an important role in tissue arrangement. Particularly in the brain where neurons are initially produced in excess numbers, cell death pathways are first freely active but later have to be attenuated during the establishment of the mature neuronal circuitry which then has to be protected from accidental cell death [5, 6].

Here, we investigated potential mechanisms underlying human neuronal longevity. Using human induced pluripotent stem cell (hiPSC)-derived neuronal cultures we analyzed the regulation of cell death pathways in different stages of neuronal maturity. By performing bulk and single-cell transcriptomics as well as immunoblotting protein analysis we show that neuronal maturation is accompanied by a multitude of adaptations that render mature neurons more resistant to a variety of cellular stressors. These adaptations take place at various key steps of the intrinsic apoptosis pathway including a downregulation of caspases, restriction of apoptosome activity, an elevated threshold for mitochondrial outer membrane permeabilization and increased potency of XIAP, the major IAP.

## Results

### Human iPSC-derived neurons develop hallmarks of maturity in vitro

**Fig. 1:**
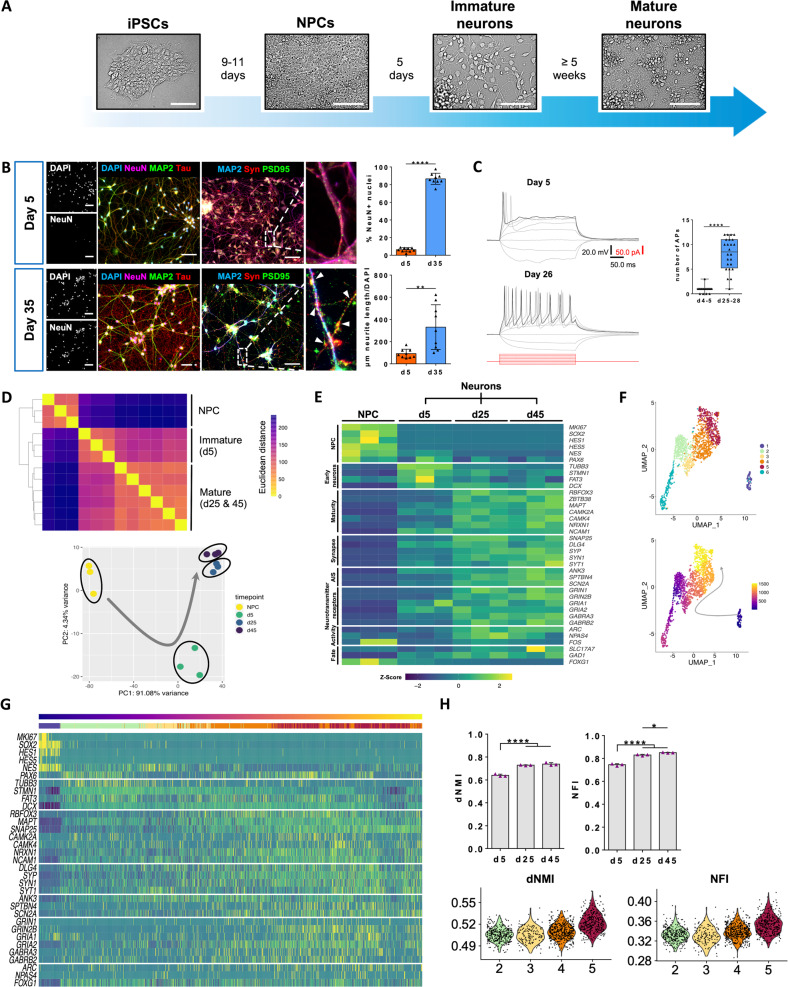
Defined maturity stage neuronal cultures from induced pluripotent stem cells. **A** Schematic representation of the differentiation protocol used for generation of human forebrain neurons from iPSCs. Brightfield images exemplify the different stages throughout differentiation. Scale bars, 100 µm. **B** Representative fluorescence microscopy images of immature neuronal cultures at day 5 and more mature neuronal cultures at day 35 of differentiation. Scale bars, 50 µm. Upper bar graph shows the quantification of the percentage of NeuN+ nuclei in d5 and d35 cultures. Graph shows mean with S.D., *P* < 0.0001, two-tailed t test. Lower bar graph shows the quantification of the degree of neurite expansion in d5 and d35 neuronal cultures. Bar graph shows mean with S.E.M. *P* = 0.0031, two-tailed t test. Every data point represents the result obtained for a single microscopy image. Three images were analyzed per differentiation and three independent differentiations were assessed. **C** Characterization of electrophysiological properties of individual neurons at d4-5 (*n* = 20 cells) and d25-28 (*n* = 24 cells) of differentiation. Shown are representative AP firing patterns of a neuron at d5 and d26 during 300 ms of depolarization. Box plot shows the number of action potentials evoked during 300 ms of depolarization (*P* < 0.0001, two-tailed Mann-Whitney U test). Each data point represents a single neuron. **D** Sample distance matrix with hierarchical clustering based on rlog normalized counts (upper panel). The degree of sample similarity is expressed by Euclidean distance. With extended time in culture, bulk transcriptomes become more similar. PCA biplot showing PC1 and PC2 based on the 500 most variable features (lower panel). Data shows biological replicates grouping together and sample separation based on time point of differentiation. Samples are color-coded by time point. Grey arrow indicates timeline of differentiation. **E** Heat map (z-scaled normalized counts) showing time point-dependent expression of canonical NPC, early neuron and more mature neuron marker genes resolved for the individual biological replicates. NPCs express the proliferation marker *MKI67* and progenitor-associated genes such as *SOX2*, *HES1*, *HES5*, *NES* and *PAX6*. Immature d5 neuronal cultures prominently expressed early neuron markers like *TUBB3*, *STMN1*, *FAT3* and *DCX*. From d25 onward, neuronal cultures expressed a wide range of neurotransmitter receptors including NMDA (*GRIN1/2B*), AMPA (*GRIA1/2*), GABA (*GABRA3, GABRAB2*) receptors and synaptic components like *SNAP25*, PSD95 (*DLG4*), *SYP*, *SYN1* and *SYT1* and the two major structural building blocks of the axon initial segment (AIS) ankyrin-G (*ANK3*) and βIV spectrin (*SPTBN4*) as well as the AIS-associated voltage-gated sodium channel Nav1.2 (*SCN2A*). **F** Dimensional reduction plot in UMAP dimensions. Seurat clustering of single cell transcriptomes reveals six distinct cell populations. Data from RNAseq was used for guided dimensional reduction of scRNAseq data (upper panel). Monocle3 pseudotime analysis based on dimensional reduction from upper panel. Grey arrow indicates progression of pseudotime with yellow data points marking cells that are most advanced along the trajectory (lower panel). **G** Heat map of marker gene expression in single cells ordered by pseudotime (z-scaled). **H** Scoring maturity of bulk neuronal cultures using the neuMatIdx R package described by He and Yu [8] The discriminating neuron maturity index (dNMI) relies on transcriptional modules best explaining the differences between immature and mature neurons. The neuron functionality index (NFI) only incorporates modules specifically enriched in mature neurons. Bar graphs show mean with S.D., one-way ANOVA with Bonferroni correction. dNMI and NFI score for the neuronal clusters identified in scRNAseq analysis showing advancing transcriptomic maturity from cluster 2 to 5.

To investigate the stress-coping competence of human neurons at different stages of maturation, we used hiPSCs to generate forebrain-type neural progenitors and neurons derived thereof (Fig. 1A and Supplementary Fig. 1A). Immature neurons (day 5) co-express the neuronal markers Tau and MAP2 without separation or compartmentalization of both. In more mature neurons (day 35) a cytoarchitectural reorganization of both markers occurred leading to the formation of a Tau+ axonal compartment and the MAP2+ dendritic arbor (Fig. 1B), indicating neuronal polarity. At d35 cultures also displayed a significantly expanded network of neurites compared to neurons at d5. At the same time, the percentage of cells expressing the maturation-associated neuronal splicing regulator NeuN increased from about 5% to >80%. Immunofluorescence examination of the pre-synaptic marker protein Synapsin and post-synaptic PSD95 revealed diffuse cytosolic distribution of Synapsin and very sparse PSD95+ punctae in day 5 neurons and abundant expression and colocalization in day 35 neurons (Fig. 1B). Upon depolarization, immature neurons (d4-5) generated none or only a single action potential (AP), whereas more mature neurons (d25-28) elicited repetitive trains of APs (Fig. 1C). More mature neurons showed a trend towards slightly more negative resting membrane potential (RMP), significantly decreased input resistance and significantly increased membrane capacitance (Supplementary Fig. 1B).

Transcriptome RNA bulk sequencing (RNAseq) of NPCs and neuronal cultures at day 5, 25 and 45 of differentiation (Supplementary Fig. 1C) revealed a high degree of similarity between biological replicates of the same time point of differentiation. With increasing time in culture, broad transcriptional changes became less pronounced. In support, principal component analysis of PC1 vs PC2 grouped NPCs and d5 neuronal cultures in distinctly separate clusters whereas samples of d25 and d45 neuronal cultures clustered more closely together (Fig. 1D). Using time course clustering of genes with similar temporal expression dynamics [7], we identified gene ontology (GO) terms related to biological processes (BP), molecular functions (MF) and cellular compartments (CC) that are characteristic for the different stages of neuronal maturation with GO terms related to DNA replication and cell proliferation enriched in NPCs, GO terms related to neuronal migration, axon extension and guidance enriched in young neurons and GO terms related to neurotransmitters and ion channels enriched in mature neuronal stages (Supplementary Fig. 1D). When investigating canonical marker genes at different stages, NPCs strongly expressed progenitor-associated markers, immature d5 neurons early neuronal markers and more mature neurons neurotransmitter receptors, synaptic components and structural building blocks of the axon initial segment (AIS). Increased network activity throughout maturation is also indicated by elevated expression of the neuronal activity-related genes *NPAS4* and *ARC*. Mature neurons expressed markers of both, excitatory and inhibitory fates (Fig. 1E). Canonical astrocyte markers were almost completely absent in our cultures (Supplementary Fig. 1E) and only single astrocytes could be detected by immunocytochemistry (Supplementary Fig. 1F).

Next, we used single-cell RNA sequencing (scRNAseq) of a mixture of neuronal cultures at different stages of differentiation ranging from day 6 to day 52 to assess transcriptomes of individual neurons with varying degrees of neuronal maturity (Supplementary Fig. 1G). Since individual neurons differentiate at a variable rate in vitro, d6 neuronal cultures may contain a small fraction of transcriptionally more mature neurons and, conversely, d52 cultures may contain single neurons of lesser maturity. To optimally sort single neurons along a gradient based on their maturity, we used our previously obtained RNAseq data of bulk cultures for guided dimensional reduction of the scRNAseq data. Based on this approach, Seurat clustering led to the identification of six distinct cell populations which were subsequently subjected to monocle3 pseudotime analysis (Fig. 1F). Marker gene analysis revealed cluster 1 at the origin of pseudotime to consist of cells expressing *MKI67*, *SOX2* and *NES* indicating NPC fate. Clusters 2 and 3 were formed by cells expressing high levels of *TUBB3*, *STMN1* and *DCX* whereas clusters 4 and 5 showed increasing expression of synaptic and maturation-associated marker genes (Fig. 1G), which is in line with immature (clusters 2-3) and mature (clusters 4-5) neurons. Cells in cluster 6 showed a strong enrichment of GO terms related to cellular stress which might have been a side-effect of the prolonged sample preparation procedure (data not shown) and was excluded from further analysis. The single-cell cluster assignment to the different stages of maturity is corroborated by pseudotime trajectory profiles of canonical hallmark genes (Supplementary Fig. 1H). To score the degree of maturity of our neuronal cultures, we calculated the discriminating neuron maturity index (dNMI) and the neuron functionality index (NFI) [8]. Both indicators are based on functional modules from literature expression data and yield a value between 0 and 1, with 1 representing a perfect maturity score. Based on our RNAseq data, the dNMI and NFI steadily increased with time of differentiation and peaked at d45 reaching 0.738 ± 0.010 and 0.851 ± 0.002 (S.D.), respectively. Of the neuronal clusters 2–5 within the scRNAseq experiment, cluster 5 averaged the highest dNMI and NFI (0.522 ± 0.013 and 0.353 ± 0.019 (S.D.)) (Fig. 1H). These data highlight the structural, functional and transcriptional maturation of the generated iPSC-derived neuronal cultures.

### Maturation-associated increase in the threshold of the intrinsic apoptosis pathway

**Fig. 2:**
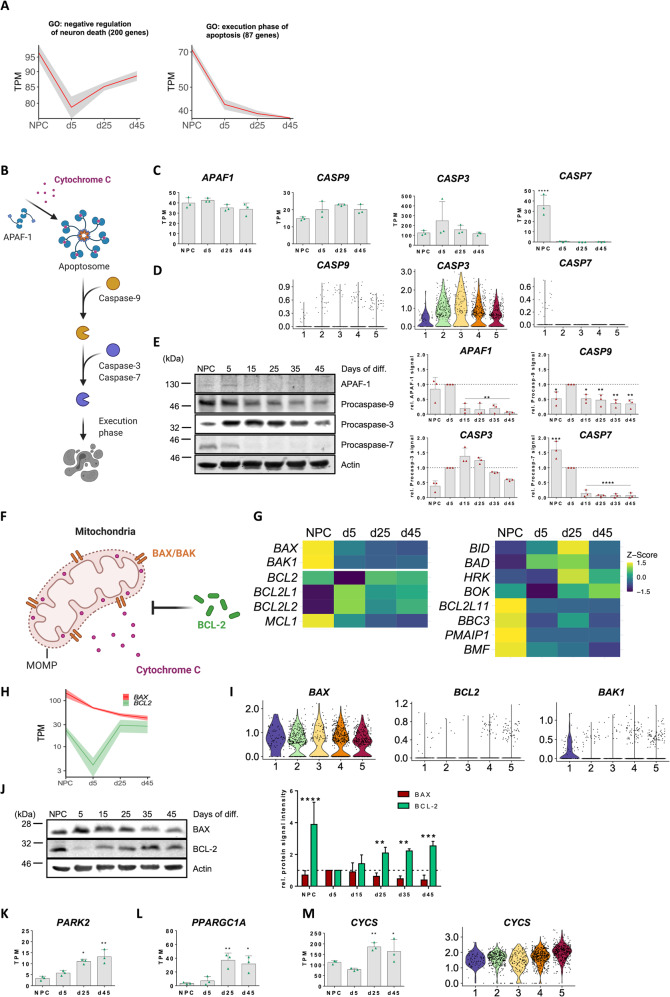
Neuronal maturation is accompanied by modulation of central components of the intrinsic apoptosis pathway. **A** shows the mean temporal expression pattern of genes belonging to the indicated GO terms based on TPM counts. Shaded area indicates S.D. **B** Schematic representation of the intrinsic apoptosis pathway. Stress stimuli trigger apoptosome formation and activation of the initiator Caspase-9 which subsequently activates downstream effector caspases. Effector caspases trigger events leading to apoptosis. **C** TPM values from RNAseq at the indicated time points of neuronal differentiation. Graphs show mean with S.D., statistical comparison to d5 neuronal cultures based on one-way ANOVA with Bonferroni correction. **D** Violin plots based on scRNAseq data tracing the different stages of neuronal maturation. **E** Representative Western blots of the essential caspases of the intrinsic apoptosis pathway and the apoptosome component APAF-1 and quantification of relative protein signal intensities based on *n* = 3 biological replicates of independent differentiations. Signals were normalized to values of d5 immature neuronal cultures (dashed line). Bar graphs show mean with S.D., one-way ANOVA with Bonferroni’s multiple comparison test. **F** Initiation of the intrinsic apoptosis pathway requires pore formation by BAX and BAK in the outer mitochondrial membrane which can be prevented by the pro-survival factor BCL-2. **G** Heat map (z-scaled normalized counts) showing expression of pro- and anti-apoptotic BCL-2 family members throughout neuronal maturation. **H** TPM-normalized mean temporal expression of *BAX* (red) and *BCL2* (green). Shaded area shows S.D. **I** Violin plots showing cluster-specific abundance of *BAX, BCL2* and *BAK1* in scRNAseq. **J** Representative Western blots showing changes in BAX and BCL-2 protein levels over the time course of neuronal maturation and quantification from *n* = 3 independent experiments. Bar graphs show mean with S.D., two-way ANOVA with Bonferroni correction. **K** Expression of *PARK2* encoding the E3 ubiquitin ligase Parkin. **L** More mature neurons show increased expression of the master regulator of mitochondrial biogenesis, *PPARGC1A*. **M** Bulk and scRNASeq analysis for Cytochrome c, which is an essential part of the mitochondrial electron transport chain. Bar graphs of K-M show mean with S.D., statistical comparison to d5 neurons using one-way ANOVA with Bonferroni multiple comparison test.

Equipped with expression data of defined maturity stages of human neurons, we next analyzed maturation-dependent adaptations in stress resistance and survival competence. We observed a drop in the mean temporal expression of 200 genes summarized under the GO term “negative regulation of neuron death” in immature d5 cultures which increased again with ongoing maturation. Conversely, mean expression of 87 genes constituting the term “execution phase of apoptosis” dropped steadily throughout maturation (Fig. 2A and Supplementary Table 1). To investigate these adaptations in more detail we assessed the expression of central proteins of the caspase cascade pathway (Fig. 2B). *APAF1* expression peaked at d5 and slightly decreased afterwards. *CASP9* expression levels appeared to remain fairly constant whereas *CASP3* expression was highest at d5 and decreased steadily thereafter. Interestingly, *CASP7* was highly expressed in NPCs but was immediately and almost completely downregulated with neuronal differentiation and maturation (Fig. 2C, D). On the protein level, APAF-1 was almost undetectable after d5 of differentiation. Additionally, protein levels of Procaspase-9 and -3 decreased over the time course of maturation. Levels of Procaspase-3 peaked around d5-d15 and then robustly decreased over time. In line with the expression data, we observed almost a complete abrogation of Procaspase-7 after d5 of neuronal maturation (Fig. 2E). For APAF-1 and Caspase-9, splicing variants with anti-apoptotic properties have been reported [9, 10]. In our cultures, however, competitive RT-PCR shows no shift towards pro-survival variants during maturation (Supplementary Fig. 2A). *CASP8* and *CASP10*, the two key caspases of the extrinsic apoptosis pathway, were barely expressed in our neuronal cultures. In addition, we noticed a significant downregulation of *CASP2* expression at later stages of neuronal maturation and a downregulation of *CASP6* in neuronal cultures in general (Supplementary Fig. 2B). We also detected a downregulation of the apoptotic chromatin condensation factor *ACIN1*, a prominent downstream target of Caspase-3 (Supplementary Fig. 2C). Thus, neuronal maturation leads to a restriction of central proteins of the caspase cascade pathway.

### Mature neurons establish a survival-promoting balance of BCL-2 family proteins

The intrinsic apoptosis pathway is initiated at the mitochondria, where the two pore-forming BCL-2 family proteins BAX and BAK cause mitochondrial outer membrane permeabilization (MOMP). MOMP leads to the release of Cytochrome c into the cytosol which is required for apoptosome formation (Fig. 2F). The action of BAX and BAK may be counteracted by pro-survival BCL-2 proteins. The balance of pro- and anti-apoptotic BCL-2 proteins decides over MOMP. In our expression data, *BAX* expression steadily declined during maturation whereas expression of *BCL2* reached a minimum at d5 but then rebounded as neurons matured (Fig. 2G–I). Additionally, we observed a strong downregulation in the expression of the second pore-forming pro-apoptotic BCL-2 protein *BAK1* at all stages of neuronal differentiation (Fig. 2G, I). Of the remaining anti-apoptotic BCL-2 members, *BCL2L1* and *BCL2L2* were more abundantly expressed in neurons whereas *MCL1* was prevalent in NPCs. The pro-apoptotic factors *BID*, *BAD* and *HRK* showed slightly higher expression in neuronal cultures than NPCs but *BCL2L11*, *BBC3*, *PMAIP1* and *BMF* were downregulated during neuronal maturation (Fig. 2G). On the protein level, BAX was highest at d5 and then continually decreased. In contrast, protein levels of the BAX antagonist BCL-2 steadily increased after barely being detectable at d5 (Fig. 2J). We also observed a significant upregulation in the expression of the ubiquitin E3 ligase Parkin (*PARK2*) during neuronal maturation (Fig. 2K). Ubiquitination of BAX and BAK by Parkin prevents their apoptotic function and leads to partial degradation [11, 12] whereas monoubiquitination by Parkin stabilizes BCL-2 [13]. As neurons mature, they also undergo a metabolic switch from a reliance on glycolysis to oxidative phosphorylation [14]. The increased number of mitochondria is reflected by a significant increase in the expression of *PPARGC1A* which encodes PGC-1α, the master regulator of mitochondrial biogenesis (Fig. 2L). Since Cytochrome c is an integral part of the electron transport chain, more mature neurons consequently contain elevated Cytochrome c levels (Fig. 2M). Therefore, a tight restriction of MOMP may be of special importance in mature neurons to prevent unsolicited release of pro-apoptotic Cytochrome c into the cytosol.

### XIAP is a central protective factor in human neurons

**Fig. 3:**
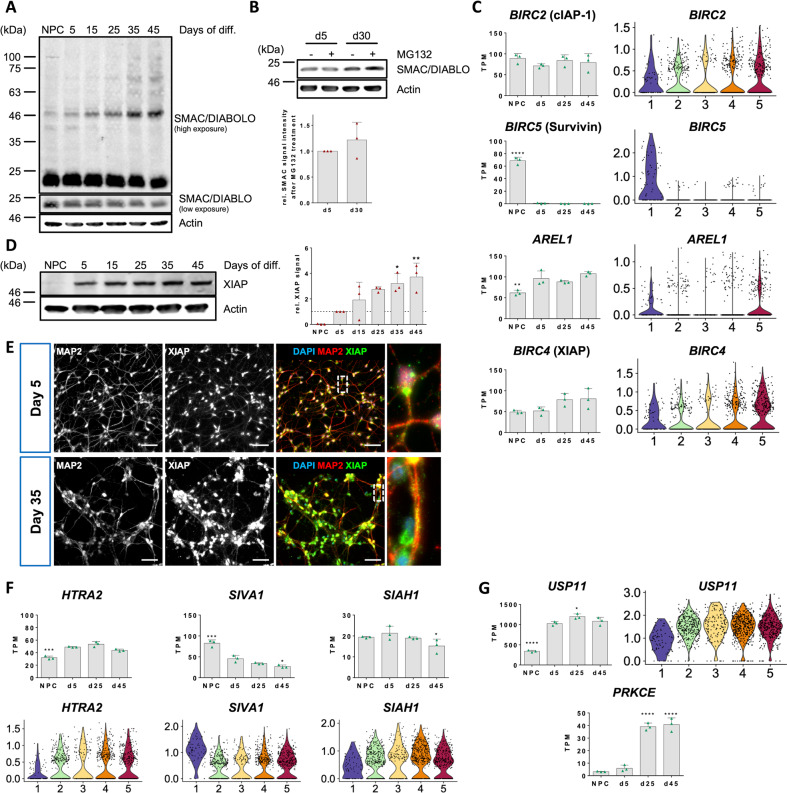
The inhibitor of apoptosis protein XIAP is a cornerstone of neuron survival competence. **A** Western blot of the pro-apoptotic mitochondrial protein SMAC/DIABLO. Top panel shows high exposure of a Western blot over the time course of neuronal maturation. Mature SMAC is detected slightly below 25 kDa. Middle panel shows band of mature SMAC from top panel at lower exposure. **B** Western blot showing inhibition of protein turnover by treatment with MG132 (5 µM) for 16 h in d5 and d30 neuronal cultures. Bar graph shows mean with S.D. of *n* = 3 experiments. **C** Expression profiles of known SMAC E3 ubiquitin ligases. Bar graphs show mean with S.D., one-way ANOVA with d5 expression as reference for statistical analysis (left row). Violin plots showing cluster-specific abundance of the same genes in scRNAseq (right row). **D** Representative Western blot of XIAP protein levels during neuronal maturation. Quantification shows relative signal intensity of XIAP Western blot band compared to d5. Bar graph shows mean with S.D., one-way ANOVA with Bonferroni correction. **E** Representative immunofluorescence images of XIAP in d5 and d35 neuronal cultures. White dashed boxes indicate picture area of the zoom-in on the right. Scale bars, 50 µm. **F** RNAseq and scRNAseq data of XIAP inhibitory proteins and (**G**) proteins involved in protecting XIAP from degradation. Bar graphs show mean with S.D., one-way ANOVA.

MOMP also enables second mitochondria-derived activator of caspases (SMAC) (also called direct IAP binding protein with low pI (DIABLO)) to leave the mitochondrial intermembrane space and enter the cytosol. SMAC/DIABLO promotes apoptosis by antagonizing IAPs which usually inhibit the activity of caspases. Interestingly, Western blotting of SMAC revealed a trend to decreased protein abundance and a gradual pattern of increased post-translational protein modification throughout neuronal maturation (Fig. 3A). Most prominently, we noticed an increase of an about 50 kDa band and a high molecular weight smear above it. This pattern closely resembles previously reported ubiquitination patterns of SMAC [15, 16]. We thus wondered, whether turnover of SMAC in mature neurons is increased. Indeed, blocking proteasomal degradation of ubiquitinated proteins by MG132 increased SMAC protein levels in mature versus immature neuronal cultures (Fig. 3B). On the RNA level, *DIABLO* did not show significant changes with neuronal maturation (Supplementary Fig. 3A) nor did we observe major differences in localization when analyzing immunofluorescence stainings (Supplementary Fig. 3B). Next, we investigated expression levels of known SMAC ubiquitin E3 ligases. Expression of *BIRC2* (cIAP-1) showed only slight variation between the different stages of neuronal maturation whereas *BIRC3* (cIAP-2) and *BIRC7* (Livin) were not detectable and expression of *BIRC1* (NAIP) was minimal. *BIRC5* (Survivin) was strongly expressed in NPCs but its expression was abrogated in neurons. However, neurons displayed increased expression of *AREL1* compared to NPCs (Fig. 3C and Supplementary Fig. 3C). Of the four neuronal scRNAseq clusters, *AREL1* was most strongly expressed in cluster 5 formed by the most mature subpopulation of neurons. *BIRC4* (XIAP) also showed a trend towards increased expression in more mature neuronal cultures in bulk RNASeq. This effect was even more pronounced on the single cell level, on which the highest XIAP mRNA levels were detected in cluster 5 (Fig. 3C). Evaluation of XIAP protein levels revealed an almost 4-fold increase of XIAP protein in day 45 compared to day 5 neurons (Fig. 3D) and this notion was supported by immunofluorescence experiments (Fig. 3E). Interestingly, NPCs possessed robust *BIRC4* expression but did not show notable amounts of XIAP protein in Western blots suggesting involvement of mechanisms for post-transcriptional downregulation of XIAP in NPCs. Finally, we investigated changes in the expression of reported XIAP antagonists aside from SMAC. Expression of the mitochondrial serine protease *HTRA2* [17] peaked at d25 and then decreased again (Fig. 3F). Furthermore, mature neurons expressed significantly less of the XIAP ubiquitinating enzymes *SIVA1* [18] and *SIAH1* [19] compared to immature neurons (Fig. 3F). *XAF1* which is capable of counteracting XIAP anti-caspase activity [20] was not expressed at any stage (Supplementary Fig. 3E). In addition, neurons possessed significantly higher expression of the ubiquitin-specific protease 11 (USP11) (Fig. 3G) which has been shown to prevent XIAP degradation via the ubiquitin proteasome system [21]. Similarly, mature neurons possessed strongly elevated levels of protein kinase Cε (PRKCE, Fig. 3G) which is able to stabilize XIAP protein levels via protective phosphorylation [22]. Together, the data hint at an increasingly strict post-translational regulation of the major IAP antagonist SMAC/DIABLO in mature neurons and implicate a critical involvement of XIAP, a concomitant decline in the expression of its antagonists and increased expression of potential XIAP stabilizers. To validate the reproducibility of our results across different clones and genetic backgrounds we analyzed transcription profiles of neurons generated from an additional clone of the same cell genetic background and from two additional genetic backgrounds which showed comparable expression intensities (Supplementary Fig. 4A). Western Blot analysis confirmed consistent regulation of apoptosis-associated and regulatory proteins in neurons from the other genetic backgrounds (Supplementary Fig. 4B). Separation of excitatory and inhibitory neurons in the scRNASeq data and comparative analysis also confirmed consistent regulation in different neurotransmitter backgrounds (Supplementary Fig. 4C). Immunocytochemical staining of XIAP, caspase7 and BCL2 in immature and mature neurons confirmed upregulation of XIAP and BCL2 and downregulation of caspase7 (Fig. 3E and Supplementary Fig. 4D). The homogeneity of expression argues for a consistent maturity-dependent regulation across the entire neuronal culture and not a selected process of a subgroup of neurons in diverse starting population.

### Mature human neurons are highly resistant to a wide range of cellular insults

**Fig. 4:**
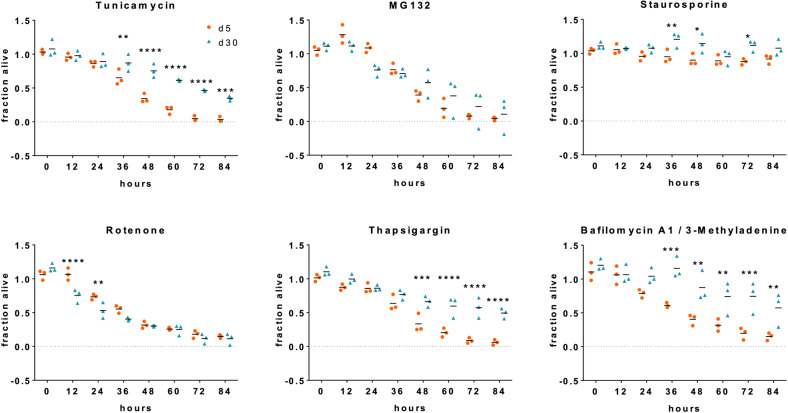
Mature human neurons display increased survival capabilities and stress resistance. Orangu™ cell viability assay determining viability of d5 and d30 neuronal cultures in the presence of the indicated stressor molecules over 84 h. Fraction of viable cells normalized to respective DMSO-treated control cells. Bar graphs show means of three independent experiments (performed in hexuplicates) and mean of the 3 experiments, two-way ANOVA with Bonferroni correction.

Equipped with such a broad range of intrinsic safeguarding mechanisms, we hypothesized more mature neurons to display a higher resistance to cellular stress in comparison to immature neurons. Thus, we exposed immature and more mature neuronal cultures to a variety of molecular stressors and evaluated their survival rates (Fig. 4). Mature neurons showed significantly higher survival rates than immature neurons when exposed to tunicamycin (inhibitor of of protein N-glycosylation) or thapsigargin (disrupts intracellular calcium homeostasis and evokes endoplasmic reticulum stress). A similar pattern was observed when interfering with the autophagic flux by combined treatment with Bafilomycin A1 and 3-methyladenine. Mature neurons also showed a tendency to cope better with MG132 (inhibitor of the ubiquitin-proteasome system) exposure but the observed differences did not reach statistical significance. Protein kinase inhibition with staurosporine did neither affect viability of immature nor mature neurons to a larger extent. Surprisingly, viability of immature and mature neurons declined similarly during long-term exposure to rotenone. The latter disrupts the electron transport chain in mitochondria leading to the generation of reactive oxygen species and oxidative stress. Due to their increased mitochondrial biogenesis and metabolic reliance on oxidative phosphorylation, mature neurons could be expected to be harmed significantly more by rotenone than immature neurons (Fig. 4). Exposure of neurons from independent genetic backgrounds to selected compounds show similar results (Supplementary Fig. 4E). These data highlight that mature neurons are protected from a wide range of potential environmental stressors. We also investigated ferroptosis as an alternative death pathway. We observed an upregulation of ‘inducers’ and a downregulation of ‘suppressors’ with neuronal maturation. Interestingly, induction of ferroptosis in immature and mature neurons by RSL3 showed a significantly increased susceptibility in young neurons indicating increased resilience of mature neurons also to this cell death pathway (Supplementary Fig. 5A).

## Discussion

Neuronal resilience against stress is an important hallmark of the human brain. In this study we tried to decipher the molecular networks resulting in human neuronal longevity and analyzed how these networks arise during development and neuronal maturation. As an experimental model we applied pure human neuronal cultures from which we generated defined stages of maturity. Using bulk and single cell RNA sequencing we highlight global transcriptional changes associated with maturation including an upregulation of ion channels, components of the synaptic machinery and functionality-associated immediate early genes. Consequently, mature cultures reach high scores with respect to neuronal maturity and functionality when benchmarking them to several human stem cell-based and primary in vitro and in vivo data (Fig. 1H and [8]). Based on these well-defined stages of neuronal cultures we investigated cytoprotective networks that human neurons become equipped with during their maturation process. We found a catalogue of adaptations which act in concert to increase the baseline threshold for induction of apoptosis at multiple steps of the involved signaling pathways.

With respect to the caspase cascade pathway, we found a general downregulation of, both, initiator and effector caspases. The downregulation is, in part, at a transcriptional level as demonstrated for CASP3, *CASP7*, *CASP6* or *CASP10*. Other caspases such as *CASP9* remain expressed at the RNA level but are downregulated at the protein level, indicating independent control mechanisms. Upstream of the caspase cascade pathway we observed a general downregulation of APAF-1 which, upon binding to Cytochrome C, forms the apoptosome. The release of Cytochrome C itself is counteracted by inhibition of MOMP. Here, several pore-forming proteins are downregulated whereas anti-apoptotic genes of the BCL-2 family are upregulated, both, on the RNA level and even more pronounced at the protein level. This idea is further supported by the transcriptional upregulation of Parkin promoting degradation of pore-forming proteins and stabilization of anti-apoptotic BCL-2. In addition, we also observed an increasing post-translational modification and elevated turnover of the major IAP antagonist SMAC/DIABLO. SMAC has been shown to be ubiquitinated by a wide range of E3 ligases [15, 16, 23, 24]. Our data suggests AREL1 and in particular XIAP as promising candidates for mediating the apparent ubiquitination of SMAC in mature human neurons. The downregulation of known XIAP inhibitors like HtrA2, SIVA-1 and SIAH-1 or, as in the case of XAF1, their complete absence coupled to an increase in XIAP levels reinforces this concept. In line with this observation, we identified USP11 and PRKCε as two potential stabilizers of XIAP protein levels, underpinning the central role of XIAP in neuronal stress resilience. Importantly, many of the genes involved in cell death regulation have additional important functions during development which complicates interpretation of expression dynamics. For instance, Caspase-3, the major effector caspase in the apoptotic cascade peaks at day 15 of differentiation and is downregulated in more mature stages. This particular pattern might reflect the involvement of this caspase in non-apoptotic functions such as axonal outgrowth, neurite branching and synaptic plasticity in this period [25, 26].

That these adaptational processes indeed result in neuronal resilience is demonstrated in our stress exposure experiments. Different brain insults such as hypoxia, protein stress or exposure to environmental toxins were simulated by exposure of our cultures to a broad range of cellular stressors. In these experiments, mature neurons showed a clear survival advantage compared to the immature stage. These safeguarding mechanisms in mature neurons may also in part explain why most neurodegenerative diseases are usually fend off for many decades and only tend to occur with advanced age. Manifestation of neurodegenerative diseases might be enabled by a weakening of the maturity-dependent protective mechanisms and a reversion back to a more immature neuronal state.

Several cell intrinsic and non-cell intrinsic pathways might contribute to stress resilience of mature human neurons. For instance, we observed a maturity-depended upregulation of NTRK2 (coding for TrKB) and NTRK3 (coding for TrKC). However, stress test experiments in the presence and absence of receptor stimulation showed comparable responses (Supplementary Fig. 5B). Among others, neuronal activity was described to mediate neuronal resilience and our neuronal cultures show a continually increase in functionality and synaptic markers with maturation (see Fig. 1). In line with this idea, electric stimulation of neuronal cultures led to an increase of the phosphorylation of AKT, a central pro-survival hub integrating many cell signaling pathways. Vice versa, blocking Na^+^-channels by tetrodotoxin or voltage gated Ca^++^-channels by nifedipine decreases AKT phosphorylation (Supplementary Fig. 5C). Additional experiments are necessary to decipher the exact role of these contributors in regulating human neuronal resilience.

In summary, we provide the first comprehensive network description of cellular adaptations in human neurons to guarantee neuronal resilience. The broad range of identified changes and adaptations suggests that no individual factor is decisive but that their redundancy provides a fail-safe mechanism for mature neurons. Importantly, our study design allows a focused view on neuron-intrinsic or neuronal network-associated cellular adaptations as other cell types are virtually absent in our cultures. In this context it has been demonstrated that other cell types such as astrocytes and microglia provide additional non-cell-autonomous protection safeguarding the human brain [27, 28].

## Materials and methods

### Human material

iPS cells were generated from healthy donors. The study was approved by the local ethics committee. All subjects gave written informed consent.

### Cell culture

IPSC lines used in this study are CIMHi001-A; registered at www.hPSCreg.eu), CIMHi068 (female, healthy donor, 25 years) and CIMHHi069 (female, healthy donor, 23 years). Cell lines are routinely checked for mycoplasma contamination. IPSCs and NPCs were cultured on dishes coated with Geltrex™ (Life Technologies). IPSCs were maintained in DMEM/F12 with glutamine and HEPES (Life Technologies) supplemented with 1% Pen/Strep, 14 ng/ml sodium selenite (Sigma-Aldrich, S5261-10G), 64 µg/ml LAAP (Sigma-Aldrich, A8960-5G), 20 µg/ml insulin (Sigma-Aldrich, 91077C-1G), 2 ng/ml TGF-β1 (Cell Guidance Systems, GFH109), 100 ng/ml FGF-2 (Cell Guidance Systems, GFH146-1000) and 11 µg/ml transferrin (Sigma-Aldrich, T3705-5G). Base medium for NPCs and neurons was DMEM/F12 with glutamine (Life Technologies) containing N2 and B27 supplement (Life Technologies), 1% Pen/Strep, 1x GlutaMAX, 1x NEAA, 800 µg/ml D-glucose. For neural induction, monolayers of iPSCs were cultivated for 8–11 days in NPC base medium supplemented with 500 nM A83-01 (Biomol GmbH, Cay9001799-25), 200 nM LDN193189 (StemCell Technologies, 72148), 2 µM XAV939 (Cell Guidance Systems, SM38-50) and 1 mM cyclopamine (Biomol GmbH, Cay11321-10). NPCs were maintained in neural induction medium without cyclopamine. Neuronal differentiation of NPCs was initiated by switching to neuronal base medium supplemented with 1.8 mM CaCl_2_, 200 µM ascorbic acid, 1 µM LM22A (Sigma-Aldrich, SML0848-25MG), 1 µM LM22B (Tocris Bioscience, 6037), 2 µM PD-0332991 (Selleck Chemicals, 1116) and 5 µM DAPT (Cell Guidance Systems, SM15-50). The day of this medium change marked day 0 of differentiation. On day 3, cultures were dissociated using TrypLE™ Express (Life Technologies) and re-plated on dishes coated with 0.1 mg/ml poly-L-lysine and 3.75 µg/ml laminin (Sigma-Aldrich, L2020-1MG). Also on day 3, the previous neuronal medium was further supplemented with 3 µM CHIR99021 (Cell Guidance Systems, SM13-50), 10 µM forskolin (Cell Guidance Systems, SM18-100), 300 µM GABA (Sigma-Aldrich, A5835-10G). At day 11, DAPT and GABA and at day 18 CHIR99021 was removed. Subsequently, neurons matured in neuronal base medium containing 1.8 mM CaCl_2_, 200 µM ascorbic acid, 2 µM PD-0332991, 1 µM LM22A and 1 µM LM22B. All experiments in this study were performed after cultivation of the cells in neuronal base media (without additional additives) for 48 hours.

### Immunocytochemistry

Cells grown on coverslips were fixed with 4% PFA (Sigma-Aldrich, 16005) and blocked in PBS containing 10% FBS (ThermoFisher, 10270-106) and 0.3% Triton X-100. Primary antibodies diluted in blocking solution were applied at 4 °C overnight. Target proteins were detected with Alexa Fluor-labelled secondary antibodies (ThermoFisher) diluted in blocking solution. Cell nuclei were stained with DAPI and coverslips were mounted on glass slides using Mowiol®/DABCO. DAPI + and NeuN+ nuclei were counted manually using Fiji. The total length of the neurite network per image was quantified using the ridge detection plugin for Fiji. The total neurite length in µm was divided by the number of cell nuclei to calculate the average neurite length per cell.

### Western blotting

Cells were lysed in 50 mM Tris-HCl (pH 7.4), 150 mM NaCl, 25 mM EDTA, 0.2% SDS with protease and phosphatase inhibitors (ThermoFisher, A32955/A32957) and sonicated. Protein yield was determined by BCA assay (ThermoFisher, 23228). Proteins were separated by tris-tricine SDS-PAGE and blotted onto 0.2 µm nitrocellulose membranes (Sigma-Aldrich, GE10600001) with a Trans-Blot® Turbo™ (BioRad Laboratories). Membranes were blocked with 5% BSA in TBST and probed overnight with primary antibody diluted in blocking solution. Primary antibodies were detected using DyLight™ IR-conjugated secondary antibodies (CellSignaling) in TBST. Proteins were visualized using a LI-COR Odyssey IR system. Signals were quantified using Fiji image analysis software and normalized by β-actin levels. Protein levels of immature d5 neuronal cultures were set to 100% and levels in NPCs and more mature neurons were stated as relative to d5 neurons. Antibodies are listed in Supplementary Table 2.

### Neuron cell viability assay

Viability of immature and mature neurons exposed to molecular stressors was determined using Orangu™ reagent (Cell Guidance Systems, OR01-500). Neuronal cultures were exposed to molecular stressors for up to 84 h. During this time, cell viability was assessed every 12 or 24 h. Neurons were seeded at 50,000 cells/well in 96-well plates. Stressor molecules were diluted in neuronal base medium (not containing any pro-survival additives; see above): 1 µl/ml DMSO, 2 mM/15 nM 3-Methyladenine/Bafilomycin A1 (VWR, CAYM13242-25/J61835.MX), 0.5 µM MG132 (Sigma-Aldrich, 0082.1), 2.5 µM Rotenone (Sigma-Aldrich, 8875.1 G), 50 nM Staurosporine (Enzo Life Sciences, LKT-S7600-M001), 1 µM Thapsigargin (Enzo Life Sciences, BML-PE180-0001), 1 µM Tunicamycin (Merck Millipore, 12819S-5MG). Each condition was measured in six technical replicates (hexuplicates) and the assay was performed on independent differentiations as indicated in the figure legends. To determine cell viability, Orangu™ reagent was added 1:10 to each well. After 2 h at 37 °C, the absorbance at 450 nm was measured with a PowerWave™ XS plate reader (BioTek). Viability of control neurons treated with DMSO was set to 100% and viability of stressed neurons was stated as relative to DMSO controls.

### Electrophysiology

Electrophysiological recordings of neurons on PLL/laminin-coated coverslips were made using an EPC9 patch clamp amplifier (HEKA Elektronik GmbH). Pipettes were pulled from 1.5 mm O.D. borosilicate glass capillaries (World Precision Instruments, 1B150F-3) with a P-97 micropipette puller (Sutter Instrument). For whole-cell recordings, pipettes were filled with intracellular solution (115 mM K-gluconate, 20 mM KCl, 10 mM Na-phosphocreatine, 4 mM Mg-ATP, 0.3 mM GTP, 0.2 mM EGTA, 10 mM HEPES at pH 7.25 and 290 mOsm) and neurons were perfused with carbogen-bubbled artificial cerebrospinal fluid (125 mM NaCl, 1 mM MgCl_2_, 2 mM CaCl_2_, 2.5 mM KCl, 10 mM D-glucose, 25 mM NaHCO_3_, 1.25 mM NaH_2_PO_4_ at 290 mOsm). Recordings were sampled at a rate of 20 kHz. First, 300 ms long voltage steps from −90 mV to +70 mV in 10 mV increments were used to record the resulting membrane currents. In current clamp mode, injection of depolarizing current pulses (duration, 300 ms) from −20 pA to 50 pA in 10 pA increments were used to evoke action potentials. Data was analyzed with Fitmaster software (HEKA Elektronik GmbH) and graphics of exemplary traces generated with IGOR Pro v6.3 (WaveMetrics).

### Bulk and single cell RNA sequencing

Detailed information on RNAseq sample preparation and data analysis can be found in the Supplemental Information.

### Quantification and statistical data analysis

Bar graphs were plotted with GraphPad Prism v6.01 and depict mean + S.D. Individual figure legends contain detailed information regarding the number of replicates and the applied statistical tests. Significance levels against respective controls are *p < 0.05, **p < 0.01, ***p < 0.001 and ****p < 0.0001.

### Electrostimulation and blocking of neuronal activity

Based on the publication of Holmes et al. 2022 [29] neurons were simulated in vitro by triggering a cell-coated cover slip at 4 evenly distributed points with electrical pulses. The electrostimulation (ES) was performed on a set up using the WCCV 3.06 software (Knowmad Technologies LLC, Tucson, AZ, USA), a Dagan potentiostat (Dagan Corporation, Minneapolis, MN, USA), and a Pine Research headstage (Pine Research Instrumentation, Durham NC, USA). Electrically evoked stimulation was performed using an insulated stainless-steel electrode (0.2 mm diameter, untwisted, Plastics One, Roanoke, VA, USA). A biphasic stimulation was applied through a linear constant current stimulus isolator (NL800A Neurolog, Medical Systems Corp, Great Neck, NY) at 60 biphasic pulses at 50 Hz, 350 μA each, 10 s in width.

Blocking of neuronal activity was performed by exposing neuronal cultures to 1 µM tetrodotoxin (TTX; Enzo Life Sciences, BML-NA120-001) or 10 µM nifedipine (Enzo Life Sciences, ALX-550-091-G005) for 30 min.

### Software

Schematic illustrations were made using BioRender.com. Fiji was used to analyze Western blots and immunofluorescence images. R and indicated packages were used for RNAseq data analysis.

## Supplementary information

Supplemental Figure S1

Supplementary Figure 2

Supplementary Figure 3

Supplimentary Figure 4

Supplementary Figure 5

Supplementary Table 1

Supplementary Table 2

Supplement Sequencing

Supplemental Material Uncropped Blots 1

Supplemental Material Uncropped Blots 2

Supplemental Material uncropped blots 3

Supplemental Material uncropped blots 4

Checklist

## Data Availability

The data discussed in this publication have been deposited in NCBI’s Gene Expression Omnibus and are accessible through GEO Series accession number GSE207986. R code that was used to analyze the data is available at https://github.com/ahoffrichter/Wilkens_et_al_2022_sequencing.
