# Supplementary material for: Diverse maturity-dependent and complementary anti-apoptotic brakes safeguard human iPSC-derived neurons from cell death
Source: Cell Death Dis. 2022 Oct 21;13(10):887. doi: 10.1038/s41419-022-05340-4 (PMC9587001; doi:10.1038/s41419-022-05340-4)
Supplement: Supplementary file 1 — Supplemental Figure S1 [file 41419_2022_5340_MOESM1_ESM.pdf]

Wilkens et al., Supplementary Figure 1 supporting Figure 1

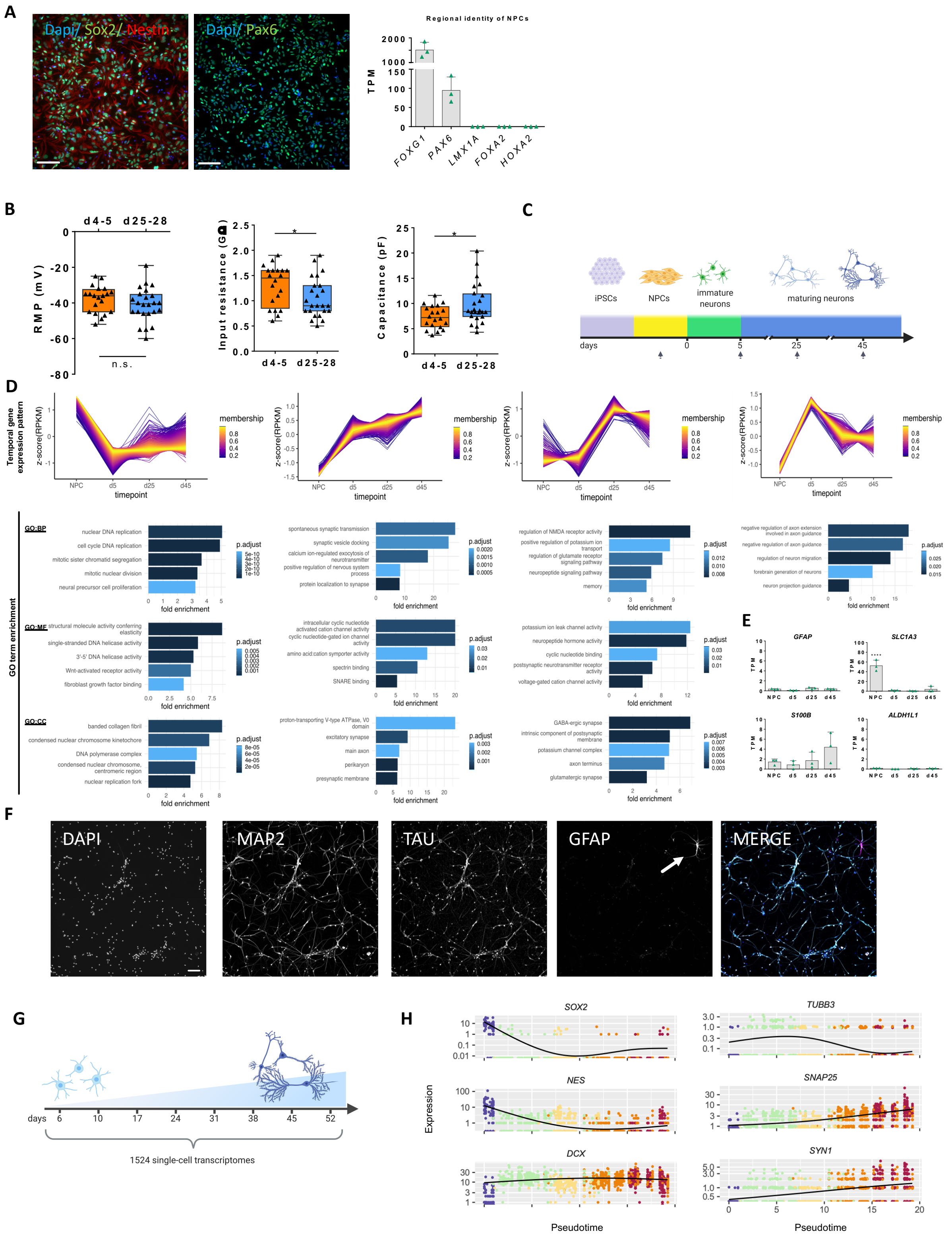

(**A**) Immunocytochemical staining showing expression of the neural progenitor markers Sox2 and Nestin as well as the transcription factor Pax6 (left panel). Scale bar: 100 µm. TPM values from RNAseq in neural progenitors showing brain regionality-associated genes. Graphs show

mean with S.D. **(B)** Box plots depicting the resting membrane potential (RMP; P = 0.2268, two-tailed t test), input resistance (P = 0.0414, two-tailed unpaired t test) and membrane capacitance (P = 0.0313, two-tailed Mann-Whitney U test). Whiskers in box plots are min-max values. Each data point represents a single neuron. **(C)** Schematic representation of the timeline of differentiation of human forebrain neurons with time points of RNAseq sample collection indicated by grey arrows. Three biological replicates of independent differentiations were analyzed for each time point. (**D**) TCseq (time course) soft clustering (cmeans) of genes based on temporal expression dynamics (z-scaled RPKM). The degree to which a gene fits to the respective cluster is indicated by its membership value with high membership values represented in yellow. Four TCseq clusters with distinct expression dynamics are shown together with the GO term enrichment analysis based on the underlying cluster-specific genes. Terms are split into three GO categories: biological process (GO:BP), molecular function (GO:MF) and cellular compartment (GO:CC). There was no significant enrichment of GO:MF or GO:CC terms associated with cluster shown in the last row. Genes strongly expressed in NPCs but downregulated in neuronal cultures (first row) represented GO terms related to DNA replication and cell proliferation. Genes with gradually increasing expression trajectories during neuronal maturation (second row) or an enrichment in more mature neuronal cultures (third row) belonged to GO terms connected to ion channel and neurotransmitter receptor regulation and signaling, synapse formation and synaptic activity. Genes that were specifically upregulated only at the beginning of neuronal differentiation and downregulated at later stages (last row) were related to processes of neuronal migration as well as negative regulation of axon extension and guidance. (**E**) Expression levels of canonical astrocyte marker genes in bulk RNAseq samples highlighting the purity of the analyzed neuronal cultures. Graphs show mean with S.D., statistical comparison to d5 neuronal cultures based on one-way ANOVA with Bonferroni correction. **(F)** Immunocytochemical stainings showing expression of the neuronal markers Map2 and Tau and the astrocytic marker GFAP. Scale bar: 100 µm. (**G**) Scheme showing collection of single cells for the generation of a maturity gradient of transcriptomes. A total of 2259 cells were sequenced. After filtering, 1524 of these cells were further analyzed. (**H**) Expression trajectories of canonical marker genes along the pseudotime axis based on normalized counts highlighting the neuronal identity of clusters 2-5.
