# Supplementary material for: Diverse maturity-dependent and complementary anti-apoptotic brakes safeguard human iPSC-derived neurons from cell death
Source: Cell Death Dis. 2022 Oct 21;13(10):887. doi: 10.1038/s41419-022-05340-4 (PMC9587001; doi:10.1038/s41419-022-05340-4)
Supplement: Supplementary file 2 — Supplementary Figure 2 [file 41419_2022_5340_MOESM2_ESM.pdf]

Wilkens et al., Supplementary Figure 2 supporting Figure 2

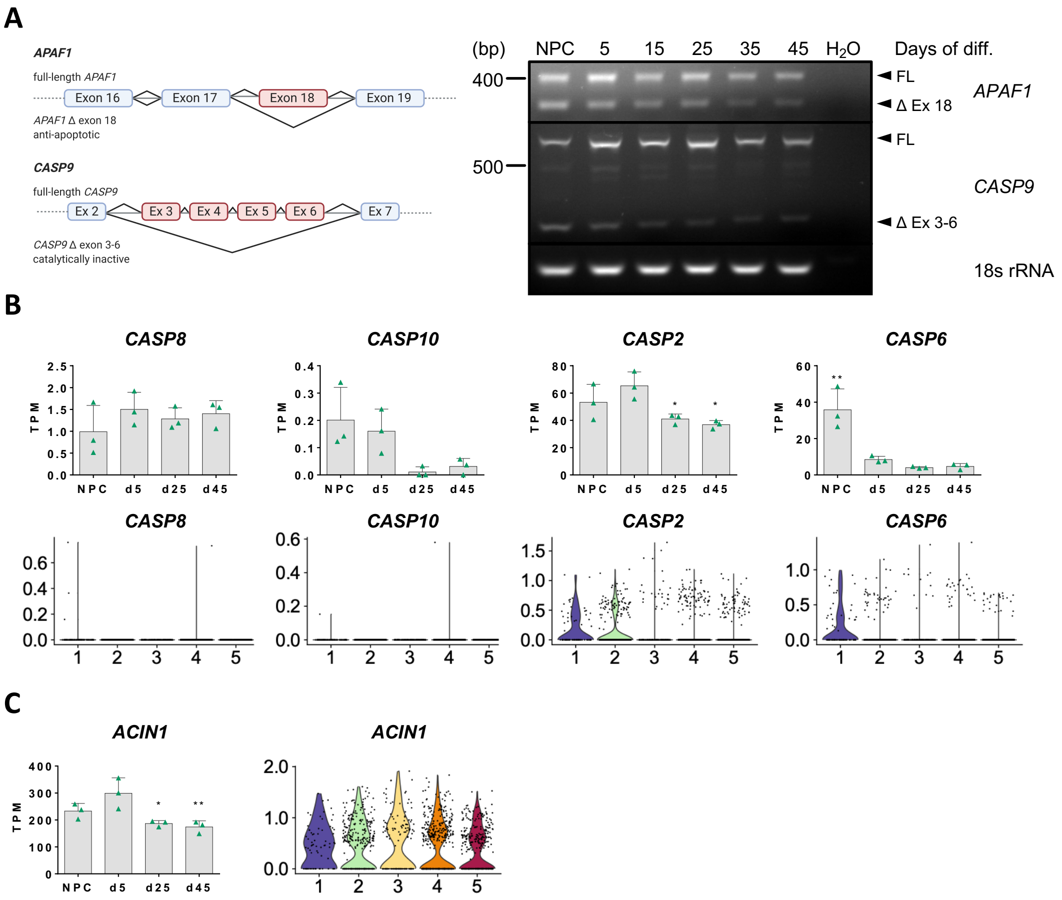

(**A**) Schematic representation showing pro- and anti-apoptotic splicing variants of *APAF1* and *CASP9*. RT-PCR for APAF1 and CASP9 splicing variants does not show a major shift between splicing products during neuronal maturation. FL: full-length. (**B**) Bulk RNAseq expression data (upper row) and violin plots showing cluster-specific scRNA data (lower row) of *CASP8* and *CASP10, CASP2* and *CASP6*. Bar graphs show mean with S.D. (**C**) Expression analysis of *ACIN1* during neuronal maturation. Bar graph with S.D., one-way ANOVA with Bonferroni multiple comparison test.
