# Supplementary material for: Diverse maturity-dependent and complementary anti-apoptotic brakes safeguard human iPSC-derived neurons from cell death
Source: Cell Death Dis. 2022 Oct 21;13(10):887. doi: 10.1038/s41419-022-05340-4 (PMC9587001; doi:10.1038/s41419-022-05340-4)
Supplement: Supplementary file 3 — Supplementary Figure 3 [file 41419_2022_5340_MOESM3_ESM.pdf]

Wilkens et al., Supplementary Figure 3 supporting Figure 3

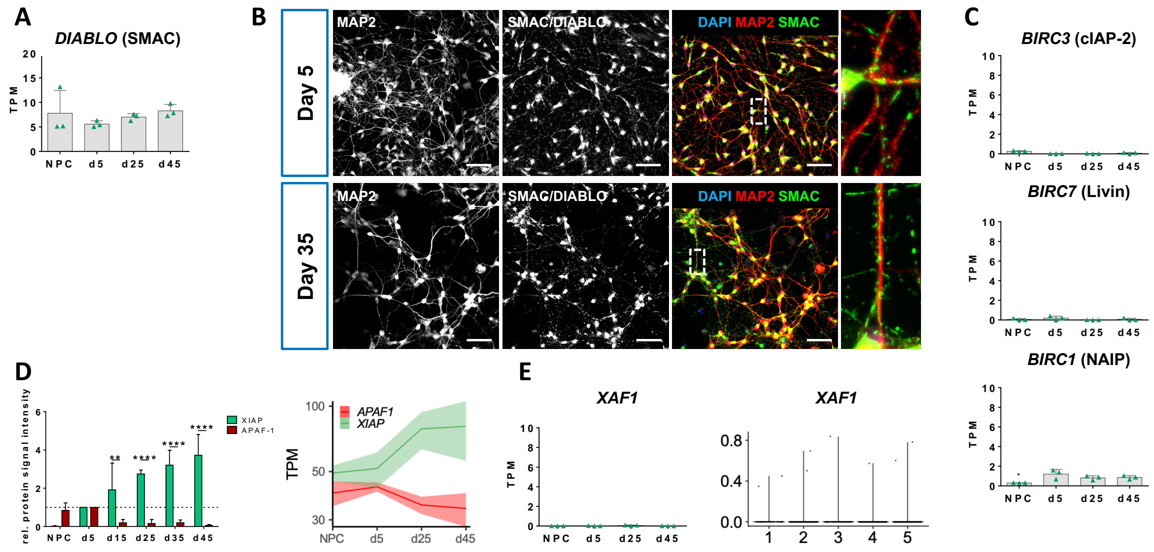

(**A**) Bar graph showing mean expression of *DIABLO* (SMAC) in TPM. (**B**) Representative immunofluorescence images of SMAC/DIABLO in d5 and d35 neuronal cultures. White dashed boxes indicate picture area of the zoom-in on the right. Scale bars, 50 µm. (**C**) Gene expression of IAP proteins that were almost completely absent during all stages of neuronal maturation. (**D**) Comparison of the development of XIAP and APAF-1 protein and mRNA levels during neuronal maturation. Bar graph shows mean with S.D. of n = 3 independent experiments, two-way ANOVA with Bonferroni correction. Mean temporal expression in TPM with shaded area indicating S.D. (**E**) Expression of the XIAP antagonist *XAF1* was mostly undetectable. Bar graphs indicate mean with S.D., one-way ANOVA with Bonferroni correction.
