# Supplementary material for: Diverse maturity-dependent and complementary anti-apoptotic brakes safeguard human iPSC-derived neurons from cell death
Source: Cell Death Dis. 2022 Oct 21;13(10):887. doi: 10.1038/s41419-022-05340-4 (PMC9587001; doi:10.1038/s41419-022-05340-4)
Supplement: Supplementary file 4 — Supplimentary Figure 4 [file 41419_2022_5340_MOESM4_ESM.pdf]

Wilkens et al., Supplementary Figure 4

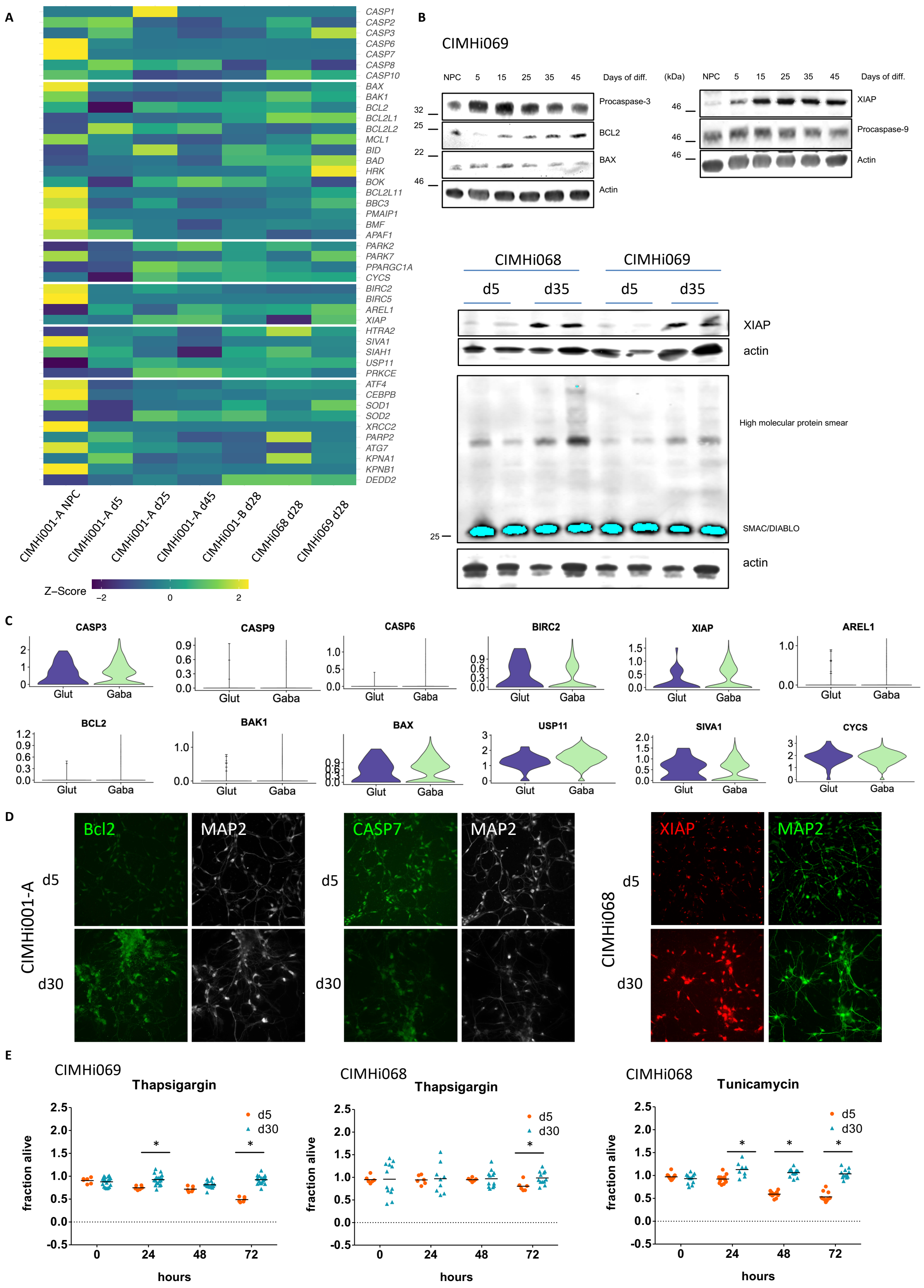

(A) Heat map (z-scaled normalized counts) expression of apoptosis-associated and regulatory genes in neurons from different genetic backgrounds. **(B)** Representative Western blots of apoptosis-associated and regulatory proteins. **(C)** Violin plots showing neuronal subtype-specific abundance of apoptosis-associated and regulatory genes in scRNAseq. Excitatory, glutamatergic neurons were selected by expression of *TBR1* and/or *SLC17A7* (coding for vGlut1). Inhibitory, GABAergic neurons were selected by expression of *GAD1* and/or *GAD2*. **(D)** Immunocytochemical stainings showing expression of caspas7, BCL2 and XIAP in immature and mature neurons. Neurons are stained with Map2. Nuclei are counterstained with DAPI. **(E)** Orangu™ cell viability assay determining viability of d5 and d30 neuronal cultures in the presence of the indicated stressor molecules over 72h. Fraction of viable cells normalized to respective DMSO-treated control cells. Bar graphs show data points from Thapsigardin CHIMi0069 d30 n=3, d5 n=1; Thapsigardin CIMHi0068 d30 n=2, d5 n=2; Tunicamycin CHIMHi0068 d30 n=2, d5 n=2 independent experiments measured in hexuplicates), two-way ANOVA with Bonferroni correction.
