# Supplementary material for: Diverse maturity-dependent and complementary anti-apoptotic brakes safeguard human iPSC-derived neurons from cell death
Source: Cell Death Dis. 2022 Oct 21;13(10):887. doi: 10.1038/s41419-022-05340-4 (PMC9587001; doi:10.1038/s41419-022-05340-4)
Supplement: Supplementary file 5 — Supplementary Figure 5 [file 41419_2022_5340_MOESM5_ESM.pdf]

Wilkens et al., Supplementary Figure 5

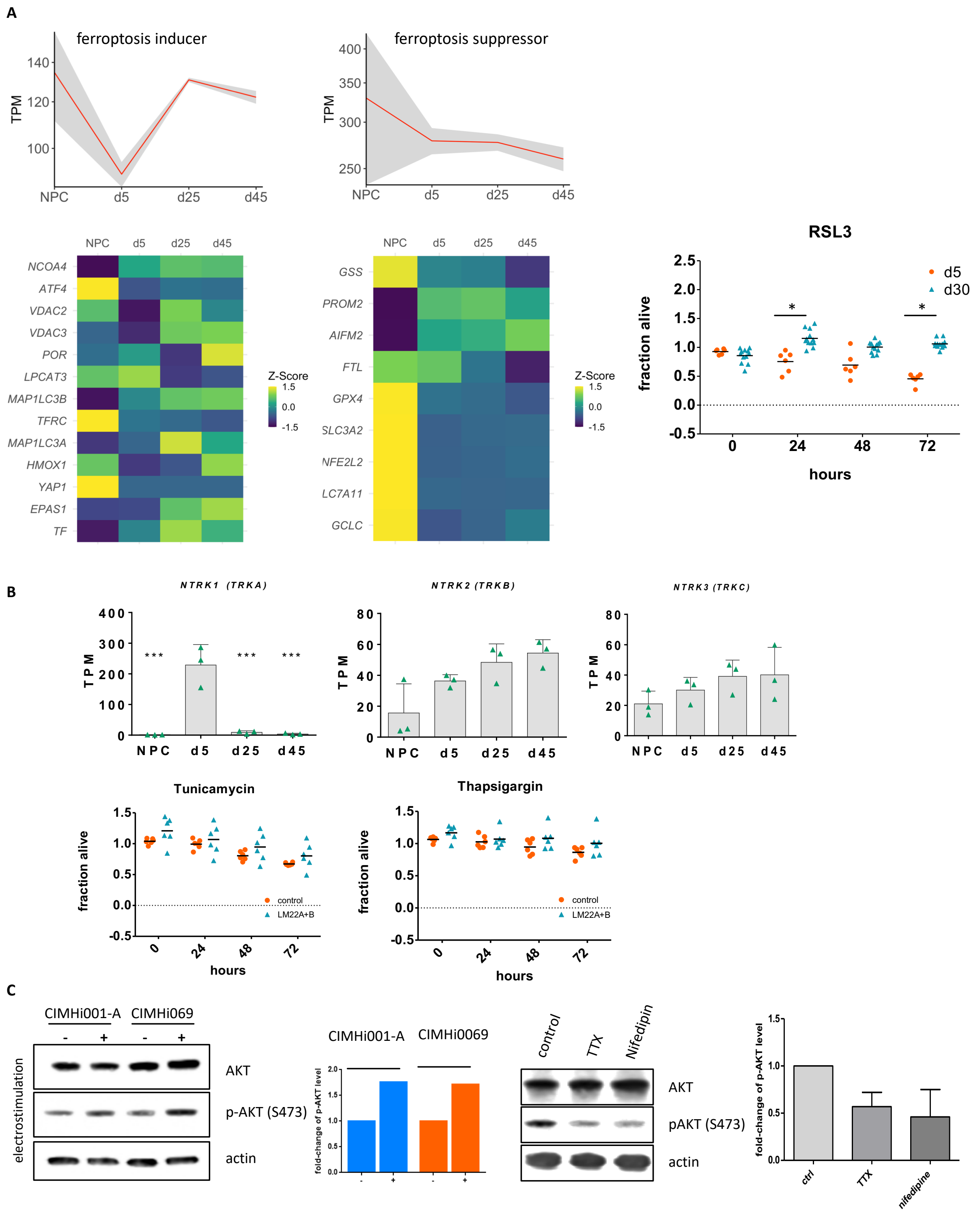

(**A**) shows the mean temporal expression pattern of genes belonging to ferroptosis inducer or ferroptosis suppressor based on a review by Kim et al. shown as TPM counts. Shaded area indicates S.D. Heat map (z-scaled normalized counts) below showing the expression of the individual genes. Orangu™ cell viability assay determining viability of d5 and d30 neuronal cultures in the presence of the ferroptosis inducer RSL3 over 72h. Fraction of viable cells

normalized to respective DMSO-treated control cells. Bar graphs show data points from 2 (day30) and 1 (day5) independent experiments measured in hexuplicates), two-way ANOVA with Bonferroni correction. **(B)** TPM values from RNAseq at the indicated time points of neuronal differentiation. Graphs show mean with S.D., statistical comparison to d5 neuronal cultures based on one-way ANOVA with Bonferroni correction. Orangu™ cell viability assay determining viability of d5 and d30 neuronal cultures in the presence or absence of the neurotrophic receptor agonists LM22A and LM22B over 72h. Fraction of viable cells normalized to respective DMSO-treated control cells. Bar graphs show data points from 1 experiment measured in hexuplicates), two-way ANOVA with Bonferroni correction. **(C)** Representative Western blots showing AKT and p-AKT with and without electrostimulation (performed in neurons from 2 independent cell lines) and quantification of p-AKT levels (Left panel). Representative Western blots showing AKT and p-AKT with and without inhibition of Na^{+}-channels by TTX of voltage gated Ca^{++}-channels by nifedipine. Quantification shows mean with S.D. from two independent experiments.

References:

Kim S-W, Kim Y, Kim SE, An J-Y. Ferroptosis-related genes in neurodevelopment and central nervous system. Biology. 2021;10:35.
