# Supplementary material for: Diverse maturity-dependent and complementary anti-apoptotic brakes safeguard human iPSC-derived neurons from cell death
Source: Cell Death Dis. 2022 Oct 21;13(10):887. doi: 10.1038/s41419-022-05340-4 (PMC9587001; doi:10.1038/s41419-022-05340-4)
Supplement: Supplementary file 6 — Supplementary Table 1 [file 41419_2022_5340_MOESM6_ESM.pdf]

Wilkens et al., Supplementary Table 1

Genes GO term: execution phase of apoptosis. Shown are mean TPM (transcripts per million) counts of NPC, d5, d25 and d45 samples respectively.

	NPC	d5	d25	d45
**AKT1**	58.798616913711	68.7828863425675	61.5374449829141	51.4795932759851
**APAF1**	39.851993153958	42.5330308465074	35.1143167067887	33.8378395819566
**BAX**	147.140498696309	69.8689033754849	49.465433442852	41.9422798560711
**BCL2L1**	106.480276369614	207.065175384392	186.823818657662	194.616888059884
**BNIP1**	25.5322897006814	18.1696141097938	20.5845239676275	24.0678957215973
**BOK**	44.2615044131665	11.8282601821447	58.9154255754894	72.8397747784746
**FOXL2**	0.179401514454971	0	0	0.033753266301559
**CASP1**	0	0	0.072085364114459	0
**CASP2**	53.2793410823237	65.3013100605175	41.0059108729131	36.8550612731878
**CASP3**	125.421732472053	248.762470797648	156.706142134371	117.534404197361
**CASP6**	35.8122375825143	8.4855346073552	4.04411779412634	4.67625722826955
**CASP7**	35.5089876788048	0.598899978105419	0.0268085414651178	0.217037246610324
**CASP8**	0.989806564313762	1.50255170503956	1.28306397426454	1.40264733775906
**CASP10**	0.201131641947237	0.160447055146435	0.0108535030005028	0.0312916721050759
**CIDEA**	0	0	0.0370339583613713	0
**DFFA**	54.4502793075115	33.2757117971579	36.1414536736491	38.2512197976794
**DFFB**	12.0522610245687	27.0253419164918	32.3035057206849	33.0423147451846
**DNASE1L3**	0	0.0635132997391823	0.02951664698796	0.0856638532475766
**DNASE2**	49.3251673196755	3.02889360802865	1.87921512500015	3.5320586530771
**ENDOG**	5.42214310909102	6.79545281507825	9.23244931129452	5.79418996719533
**FAP**	2.1635382804432	0	0.00979392987080677	0.0140594427819003
**GCG**	0	0	0	0.030594303793135
**GPER1**	0.481303468272173	0.252085025236958	0.530449404490438	0.614944951434039
**HMGB1**	141.84960756213	65.2668404312152	97.3386179023686	74.408308918806
**HMGB2**	1641.07074575682	26.8355827102442	19.0307495526962	14.7530388010544
**HSF1**	65.8299355286683	60.3584256970446	58.6978804092779	49.9301581199801
**IL6**	0.094986276807637	0	0	0
**KPNA1**	53.8829581590826	69.0470000296952	63.4054289014992	65.9542686919481
**KPNB1**	411.048600610315	163.692008300072	107.146755745658	138.708292447178
**PAK2**	136.813599869726	185.572418494764	119.620892324209	97.6674626992394
**PTGIS**	77.2668000257159	7.98487942594874	15.683400703278	16.3806427364942
**RFPL1**	0	0.058259929813536	0.156094065829479	0.114446211378157
**RPS3**	589.59790813089	229.252634898907	224.226791029923	217.50390603444
**TOP2A**	807.141738010833	7.26206497754547	0.756164048726427	1.239780532307
**TP53**	190.557566137818	20.2305181907751	15.9698457588155	12.9275269707556
**TP53BP2**	107.730115070348	64.6236458611407	37.1118892576643	37.4600665017651
**FZD3**	172.264997231414	113.366583442251	87.0202915511173	66.037535244525
**STK24**	128.93975663536	60.0700231422687	63.063092441988	62.7289460468754

**MADD**	35.0813389461352	167.170647366803	212.680041516113	201.329627871058
**AIFM1**	127.451275356923	91.0431404855414	82.9399507573842	96.6198920385858
**EXOG**	31.3240675210655	43.1421129170962	32.1316299143201	33.3652689085092
**DNM1L**	98.5517226073698	162.118200912717	172.897737247675	169.200373556627
**DLC1**	1.79661994973484	2.54769511713756	16.2432253528028	14.1291843177292
**2,00 ERN**	0.722065124499736	0.831797670299822	0.92987708948308	0.604314742560176
**BLCAP**	67.1606714132208	245.895075763141	343.106434211431	368.258424259759
**CAPN10**	30.1216465514839	44.8252032280882	36.6311472653986	31.8940019449546
**SIRT2**	19.0980358697405	99.2635919029276	103.020001804546	100.184008338108
**ACIN1**	232.932363956744	298.831387773722	186.711337340972	173.952728760435
**DICER1**	132.060475307055	119.455118400859	106.058673659349	99.7230564775546
**BBC3**	62.4537754262992	26.546393428725	49.8018939225043	29.3173997563894
**CIDEB**	0	0	0	0
**HTRA2**	31.8477566545707	49.0019764774194	53.5933487992735	43.7529570726675
**CECR2**	65.9270589840829	43.8281787233115	16.6237721882606	13.2772925309642
**PAM16**	10.0317432131753	8.92331246079149	9.19222966925863	10.5127718055951
**XKR8**	14.1213607366164	6.57758680170636	6.2695800136347	5.44538134269417
**TAOK1**	73.9937779529399	98.1708858320561	100.386952497126	100.524167497836
**DNASE2B**	0.0524894844005983	13.2183004191008	5.54664490616637	4.13356114763579
**CIDEC**	0	0.107056459446031	0	0.139470168362064
**NMNAT1**	9.6987053779256	8.10262766274878	9.32028465214944	9.3964941657491
**COLEC11**	0.323579704144422	0.403695137171759	1.46875371074244	1.06219881083025
**ZC3H12A**	4.3608396227562	2.05254309547929	1.30525663598113	0.912773205967685
**RNF34**	44.0136113440791	49.5977400297879	36.2581357332926	37.6569596087733
**CDK5RAP3**	150.011636108186	199.764699390968	136.811521706192	144.42545121562
**SHARPIN**	58.1713318811807	56.8753446424503	65.9217796624265	53.8250794435996
**XKR4**	0.579447139874343	50.1377271230549	55.2865944465534	51.4985635279532
**RFFL**	7.08186133848658	9.37050956971252	9.07341665531998	9.33317711892576
**ACVR1C**	0.266856188233978	0.595030127451307	1.32297684857033	1.47511887849988
**AIFM3**	1.15503071710342	0.691393524777696	0.873064140828682	1.36591936057036
**DEDD2**	34.5281302053323	54.3263283226876	67.1871067331904	58.022522335807
**XKR6**	6.56267209537233	4.79130916252993	7.71062384683937	6.7997043847915
**XKR7**	2.3219393743453	86.8631850111236	63.856882827646	63.0128619308953
**XKR9**	1.24420918418609	0.271412516799883	0.465102713734359	1.54859492349573
**ST20**	15.3099063693002	27.982692570755	22.6037132100555	18.3096266239782
**MIR146A**	0.0373037514740697	0.044441424643233	0.146409654984475	0.114497674070378
**MTRNR2L7**	0	0	0	0
**MTRNR2L1**	0.331159334082701	0.317624400709171	0.342880330852573	0.122640446055218
**MTRNR2L2**	1.11032659043545	0.726216705681102	1.09332856728289	1.28909363700467
**MTRNR2L3**	0.366298851288538	0.34455046687283	0.620846657305695	0.94800077960395
**MTRNR2L4**	0	0	0.0669901834263363	0.384776119452432
**MTRNR2L5**	0.049359362933775	0.26664762328735	0.22518544111097	0.401454760375849
**MTRNR2L6**	0.0621874530400183	0	0.0569902666190877	0.150836049246873
**MTRNR2L8**	0.864915861348088	0.718915064956125	0.977895450964007	0.964761616867923

**MTRNR2L9**	0	0.165383026567457	0	0.18682785161603
**MTRNR2L10**	0	0	0	0
**MTRNR2L11**	0.0499191230510843	0	0	0
**MTRNR2L12**	2.42190186317619	1.92432420943394	2.52634891743363	2.95162253042699
**MTRNR2L13**	0	0	0	0

Genes GO term: negative regulation neuron death. Shown are mean TPM (transcripts per million) counts of NPC, d5, d25 and d45 samples respectively.

	NPC	d5	d25	d45
**AKT1**	58.798616913711	68.7828863425675	61.5374449829141	51.4795932759851
**APAF1**	39.851993153958	42.5330308465074	35.1143167067887	33.8378395819566
**BAX**	147.140498696309	69.8689033754849	49.465433442852	41.9422798560711
**BCL2L1**	106.480276369614	207.065175384392	186.823818657662	194.616888059884
**BNIP1**	25.5322897006814	18.1696141097938	20.5845239676275	24.0678957215973
**BOK**	44.2615044131665	11.8282601821447	58.9154255754894	72.8397747784746
**FOXL2**	0.179401514454971	0	0	0.033753266301559
**CASP1**	0	0	0.072085364114459	0
**CASP2**	53.2793410823237	65.3013100605175	41.0059108729131	36.8550612731878
**CASP3**	125.421732472053	248.762470797648	156.706142134371	117.534404197361
**CASP6**	35.8122375825143	8.4855346073552	4.04411779412634	4.67625722826955
**CASP7**	35.5089876788048	0.598899978105419	0.0268085414651178	0.217037246610324
**CASP8**	0.989806564313762	1.50255170503956	1.28306397426454	1.40264733775906
**CASP10**	0.201131641947237	0.160447055146435	0.0108535030005028	0.0312916721050759
**CIDEA**	0	0	0.0370339583613713	0
**DFFA**	54.4502793075115	33.2757117971579	36.1414536736491	38.2512197976794
**DFFB**	12.0522610245687	27.0253419164918	32.3035057206849	33.0423147451846
**DNASE1L3**	0	0.0635132997391823	0.02951664698796	0.0856638532475766
**DNASE2**	49.3251673196755	3.02889360802865	1.87921512500015	3.5320586530771
**ENDOG**	5.42214310909102	6.79545281507825	9.23244931129452	5.79418996719533
**FAP**	2.1635382804432	0	0.00979392987080677	0.0140594427819003
**GCG**	0	0	0	0.030594303793135
**GPER1**	0.481303468272173	0.252085025236958	0.530449404490438	0.614944951434039
**HMGB1**	141.84960756213	65.2668404312152	97.3386179023686	74.408308918806
**HMGB2**	1641.07074575682	26.8355827102442	19.0307495526962	14.7530388010544
**HSF1**	65.8299355286683	60.3584256970446	58.6978804092779	49.9301581199801
**IL6**	0.094986276807637	0	0	0
**KPNA1**	53.8829581590826	69.0470000296952	63.4054289014992	65.9542686919481
**KPNB1**	411.048600610315	163.692008300072	107.146755745658	138.708292447178
**PAK2**	136.813599869726	185.572418494764	119.620892324209	97.6674626992394
**PTGIS**	77.2668000257159	7.98487942594874	15.683400703278	16.3806427364942
**RFPL1**	0	0.058259929813536	0.156094065829479	0.114446211378157
**RPS3**	589.59790813089	229.252634898907	224.226791029923	217.50390603444
**TOP2A**	807.141738010833	7.26206497754547	0.756164048726427	1.239780532307

**TP53**	190.557566137818	20.2305181907751	15.9698457588155	12.9275269707556
**TP53BP2**	107.730115070348	64.6236458611407	37.1118892576643	37.4600665017651
**FZD3**	172.264997231414	113.366583442251	87.0202915511173	66.037535244525
**STK24**	128.93975663536	60.0700231422687	63.063092441988	62.7289460468754
**MADD**	35.0813389461352	167.170647366803	212.680041516113	201.329627871058
**AIFM1**	127.451275356923	91.0431404855414	82.9399507573842	96.6198920385858
**EXOG**	31.3240675210655	43.1421129170962	32.1316299143201	33.3652689085092
**DNM1L**	98.5517226073698	162.118200912717	172.897737247675	169.200373556627
**DLC1**	1.79661994973484	2.54769511713756	16.2432253528028	14.1291843177292
**2,00 ERN**	0.722065124499736	0.831797670299822	0.92987708948308	0.604314742560176
**BLCAP**	67.1606714132208	245.895075763141	343.106434211431	368.258424259759
**CAPN10**	30.1216465514839	44.8252032280882	36.6311472653986	31.8940019449546
**SIRT2**	19.0980358697405	99.2635919029276	103.020001804546	100.184008338108
**ACIN1**	232.932363956744	298.831387773722	186.711337340972	173.952728760435
**DICER1**	132.060475307055	119.455118400859	106.058673659349	99.7230564775546
**BBC3**	62.4537754262992	26.546393428725	49.8018939225043	29.3173997563894
**CIDEB**	0	0	0	0
**HTRA2**	31.8477566545707	49.0019764774194	53.5933487992735	43.7529570726675
**CECR2**	65.9270589840829	43.8281787233115	16.6237721882606	13.2772925309642
**PAM16**	10.0317432131753	8.92331246079149	9.19222966925863	10.5127718055951
**XKR8**	14.1213607366164	6.57758680170636	6.2695800136347	5.44538134269417
**TAOK1**	73.9937779529399	98.1708858320561	100.386952497126	100.524167497836
**DNASE2B**	0.0524894844005983	13.2183004191008	5.54664490616637	4.13356114763579
**CIDEC**	0	0.107056459446031	0	0.139470168362064
**NMNAT1**	9.6987053779256	8.10262766274878	9.32028465214944	9.3964941657491
**COLEC11**	0.323579704144422	0.403695137171759	1.46875371074244	1.06219881083025
**ZC3H12A**	4.3608396227562	2.05254309547929	1.30525663598113	0.912773205967685
**RNF34**	44.0136113440791	49.5977400297879	36.2581357332926	37.6569596087733
**CDK5RAP3**	150.011636108186	199.764699390968	136.811521706192	144.42545121562
**SHARPIN**	58.1713318811807	56.8753446424503	65.9217796624265	53.8250794435996
**XKR4**	0.579447139874343	50.1377271230549	55.2865944465534	51.4985635279532
**RFFL**	7.08186133848658	9.37050956971252	9.07341665531998	9.33317711892576
**ACVR1C**	0.266856188233978	0.595030127451307	1.32297684857033	1.47511887849988
**AIFM3**	1.15503071710342	0.691393524777696	0.873064140828682	1.36591936057036
**DEDD2**	34.5281302053323	54.3263283226876	67.1871067331904	58.022522335807
**XKR6**	6.56267209537233	4.79130916252993	7.71062384683937	6.7997043847915
**XKR7**	2.3219393743453	86.8631850111236	63.856882827646	63.0128619308953
**XKR9**	1.24420918418609	0.271412516799883	0.465102713734359	1.54859492349573
**ST20**	15.3099063693002	27.982692570755	22.6037132100555	18.3096266239782
**MIR146A**	0.0373037514740697	0.044441424643233	0.146409654984475	0.114497674070378
**MTRNR2L7**	0	0	0	0
**MTRNR2L1**	0.331159334082701	0.317624400709171	0.342880330852573	0.122640446055218
**MTRNR2L2**	1.11032659043545	0.726216705681102	1.09332856728289	1.28909363700467
**MTRNR2L3**	0.366298851288538	0.34455046687283	0.620846657305695	0.94800077960395

**MTRNR2L4**	0	0	0.0669901834263363	0.384776119452432
**MTRNR2L5**	0.049359362933775	0.26664762328735	0.22518544111097	0.401454760375849
**MTRNR2L6**	0.0621874530400183	0	0.0569902666190877	0.150836049246873
**MTRNR2L8**	0.864915861348088	0.718915064956125	0.977895450964007	0.964761616867923
**MTRNR2L9**	0	0.165383026567457	0	0.18682785161603
**MTRNR2L10**	0	0	0	0
**MTRNR2L11**	0.0499191230510843	0	0	0
**MTRNR2L12**	2.42190186317619	1.92432420943394	2.52634891743363	2.95162253042699
**MTRNR2L13**	0	0	0	0
