# Supplementary material for: Diverse maturity-dependent and complementary anti-apoptotic brakes safeguard human iPSC-derived neurons from cell death
Source: Cell Death Dis. 2022 Oct 21;13(10):887. doi: 10.1038/s41419-022-05340-4 (PMC9587001; doi:10.1038/s41419-022-05340-4)
Supplement: Supplementary file 7 — Supplementary Table 2 [file 41419_2022_5340_MOESM7_ESM.pdf]

Wilkens et al., Supplementary Table 2

Antibodies for WB and ICC used in this study.

Antibody/target	Manufacturer	Catalogue number
AKT	CellSignaling	4691S
Phosphor-AKT (Ser473)	CellSignaling	9271S
β-actin	CellSignaling	3700S
β-actin	CellSignaling	4970S
APAF-1	CellSignaling	8968S
BAX	CellSignaling	5023T
BCL-2	CellSignaling	4223T
Caspase-3	CellSignaling	9662S
Caspase-7	CellSignaling	12827S
Caspase-9	CellSignaling	9508S
MAP2	BioLegend	822501
NeuN	CellSignaling	24307S
PARP-1	CellSignaling	9542S
PSD95	BioLegend	810401
SMAC/DIABLO	CellSignaling	15108S
Synapsin	BioLegend	853701
Tau	Synaptic Systems	314004
XIAP	CellSignaling	2045S
Alexa 488 anti-chicken	ThermoFisher	A11039
Alexa 568 anti-guinea pig	ThermoFisher	A11075
Alexa 488 anti-mouse	ThermoFisher	A11001
Alexa 568 anti-mouse	ThermoFisher	A11004

Alexa 488 anti-rabbit	ThermoFisher	A11008
Alexa 555 anti-rabbit	ThermoFisher	A21428
Alexa 647 anti-rabbit	ThermoFisher	A21244
DyLight™ 680 anti-mouse	CellSignaling	5470S
DyLight™ 680 anti-rabbit	CellSignaling	5366S
DyLight™ 800 anti-mouse	CellSignaling	5257S
DyLight™ 800 anti-rabbit	CellSignaling	5151S
