# Supplementary material for: Diverse maturity-dependent and complementary anti-apoptotic brakes safeguard human iPSC-derived neurons from cell death
Source: Cell Death Dis. 2022 Oct 21;13(10):887. doi: 10.1038/s41419-022-05340-4 (PMC9587001; doi:10.1038/s41419-022-05340-4)
Supplement: Supplementary file 8 — Supplement Sequencing [file 41419_2022_5340_MOESM8_ESM.pdf]

Bulk RNA sequencing (RNAseq)

Three biological replicates of independent differentiations of NPCs, d5, d25 and d45 from cell line CIMH001-A, two independent differentiations of d28 from cell line CIMHi001-B, one differentiation of cell line CIMHi0069 and two independent differentiations of cell line CIMHi0068 were analyzed. The quality of extracted RNA was assessed using the RNA 6000 Nano kit (Agilent Technologies, 5067-1511). Quality of RNA samples was assessed using a Bioanalyzer 2100 (Agilent Technologies) and samples with RIN values ≥ 7.5 were sent to the High Throughput Sequencing Unit of the Genomics & Proteomics Core Facility, German Cancer Research Center (DKFZ) to be processed. Libraries were prepared with the TruSeq Stranded protocol (Illumina) and sequenced to 50 bp single reads on one lane on the Illumina HiSeq 4K platform. Sequencing data was run through an RNAseq processing workflow by the Omics IT and Data Management Core Facility, German Cancer Research Center (DKFZ). Total counts per feature were imported to R (R Core Team 2020) and analyzed using DESeq2 (Love et al. 2014). All features without any counts were removed, for differential testing with DESeq2 the formula "~timepoint" was used.

Euclidean distance between samples was generated from rlog transformed counts using the stats package (R Core Team 2020). The sample distance plot was generated using the pheatmap package (Kolde 2019) with the viridis (Garnier 2018) plasma color scale. Principal component analysis (PCA) was performed on rlog transformed counts with the top 500 variable features using prcomp (R Core Team 2020). Principal components (PC) 1 and 2 are shown in the plot.

The TCseq package (Wu and Gu 2020) was used to perform time course sequencing analysis in which temporal patterns in gene expression are detected and genes, with similar temporal expression patterns are grouped. For clustering, the cmeans algorithm was used with k=12. Features from specific gene clusters were used to perform gene ontology (GO) enrichment analysis using enrichGO from the clusterProfiler package (Yu et al. 2012). The organism database used for this analysis is org.Hs.eg.db (Carlson 2020). Heatmaps for RNAseq expression data show z-scaled DESeq2 normalized counts.

For detailed information about the data analysis pipeline in R refer to (https://github.com/ahoffrichter/Wilkens_et_al_2022_sequencing).

Single cell RNA sequencing (scRNAseq)

Neuronal cultures at different stages of differentiation were dissociated into single cells with TrypLE™ Express (Life Technologies, 11360039). An equal number of cells from each time point were mixed to yield a unified cell suspension. cDNA library preparation was done at the single-cell Open Lab at the German Cancer Research Center (DKFZ; Heidelberg, Germany) according to the 10x Genomics Chromium single cell 3’ library & gel bead kit v2 chemistry user guide (10x Genomics, PN-120267). The prepared cDNA library was processed by the High Throughput Sequencing Unit of the Genomics & Proteomics Core Facility of the German Cancer Research Center (DKFZ). The library was sequenced on one lane on the Illumina HiSeq 4K platform with a protocol specific for 10x scRNA libraries (paired-end 26+74). Fastq files were parsed to cellranger (10x Genomics) count in order to generate a count matrix. FastQC was used for general sequencing quality control (Andrews et al. 2015). If not stated otherwise, data analysis was performed using the Seurat package (Stuart et al. 2019) in R. Count matrix

was filtered with following parameters: Any feature that was expressed in less than three cells was removed from the analysis. Any cell with less than 2000 expressed features, more than 10% mitochondrial genes expressed, or less than 5000 total UMI counts was removed from further analysis. The data was normalized using sctransform.

Differentially expressed genes (in total 2671) from RNAseq for time points NPC, d5, d25, d45, or d25&d45 were used as variable features for PCA. Further dimensional reduction was performed using UMAP with dims=1:30. Shared nearest neighbor graph was constructed with dims=1:50 and k.param=30. Clusters were generated with resolution=1.

The pseudotime trajectory was calculated with the monocle3 package (Trapnell et al. 2014; Qiu et al. 2017a, 2017b; Cao et al. 2014). Calculations for the Neuron Maturity Index were performed using the neuMatIdx package (He and Yu 2018) using normalized counts from the Seurat object. Violin plots were generated with ggplot2 and show normalized expression values. For detailed information about the data analysis pipeline in R refer to (https://github.com/ahoffrichter/Wilkens_et_al_2022_sequencing).

References

Andrews S, Krueger F, Segonds-Pichon A, Biggins L, Krueger C, Wingett S. 2015. FastQC: a quality control tool for high throughput sequence data. http://www.bioinformatics.babraham.ac.uk/projects/fastqc (Accessed December 10, 2020).

Cao J, Spielmann M, Qiu X, Huang X, Ibrahim DM, Hill AJ, Zhang F, Mundlos S, Christiansen L, Steemers FJ, et al. 2014. The dynamics and regulators of cell fate decisions are revealed by pseudo-temporal ordering of single cells. *Nat Biotechnol* **32**: 381–86.

Carlson M. 2020. org.Hs.eg.db: Genome wide annotation for Human.

Garnier S. 2018. viridis: Default Color Maps from “matplotlib.” https://cran.r-project.org/package=viridis.

He Z, Yu Q. 2018. Identification and characterization of functional modules reflecting

transcriptome transition during human neuron maturation. *BMC Genomics* **19**: 1–11.

Kolde R. 2019. pheatmap: Pretty Heatmaps. https://cran.r-project.org/package=pheatmap.

Love MI, Huber W, Anders S. 2014. Moderated estimation of fold change and dispersion for RNA-seq data with DESeq2. *Genome Biol* **15**: 550.

Qiu X, Hill A, Packer J, Lin D, Ma Y-A, Trapnell C. 2017a. Single-cell mRNA quantification and differential analysis with Census. *Nat Methods* **14**: 309–15.

Qiu X, Mao Q, Tang Y, Wang L, Chawla R, Pliner H, Trapnell C. 2017b. Reverse graph embedding resolves complex single-cell developmental trajectories. *Nat Methods* **14**: 979–82.

R Core Team. 2020. R: A Language and Environment for Statistical Computing. https://www.r-project.org/.

Stuart T, Butler A, Hoffman P, Hafemeister C, Papalexi E, III WMM, Hao Y, Stoeckius M, Smibert P, Satija R. 2019. Comprehensive Integration of Single-Cell Data. *Cell* **177**: 1888–1902.

Trapnell C, Cacchiarelli D, Grimsby J, Pokharel P, Li S, Morse M, Lennon NJ, Livak KJ, Mikkelsen TS, Rinn JL. 2014. The dynamics and regulators of cell fate decisions are revealed by pseudo-temporal ordering of single cells. *Nat Biotechnol* **32**: 381–86.

Wu M, Gu L. 2020. TCseq: Time course sequencing data analysis.

Yu G, Wang L-G, Han Y, He Q-Y. 2012. clusterProfiler: an R package for comparing biological themes among gene clusters. *Omi A J Integr Biol* **16**: 284–287.
