# Supplementary figures and images for: Diverse maturity-dependent and complementary anti-apoptotic brakes safeguard human iPSC-derived neurons from cell death
Source: Cell Death Dis. 2022 Oct 21;13(10):887. doi: 10.1038/s41419-022-05340-4 (PMC9587001; doi:10.1038/s41419-022-05340-4)

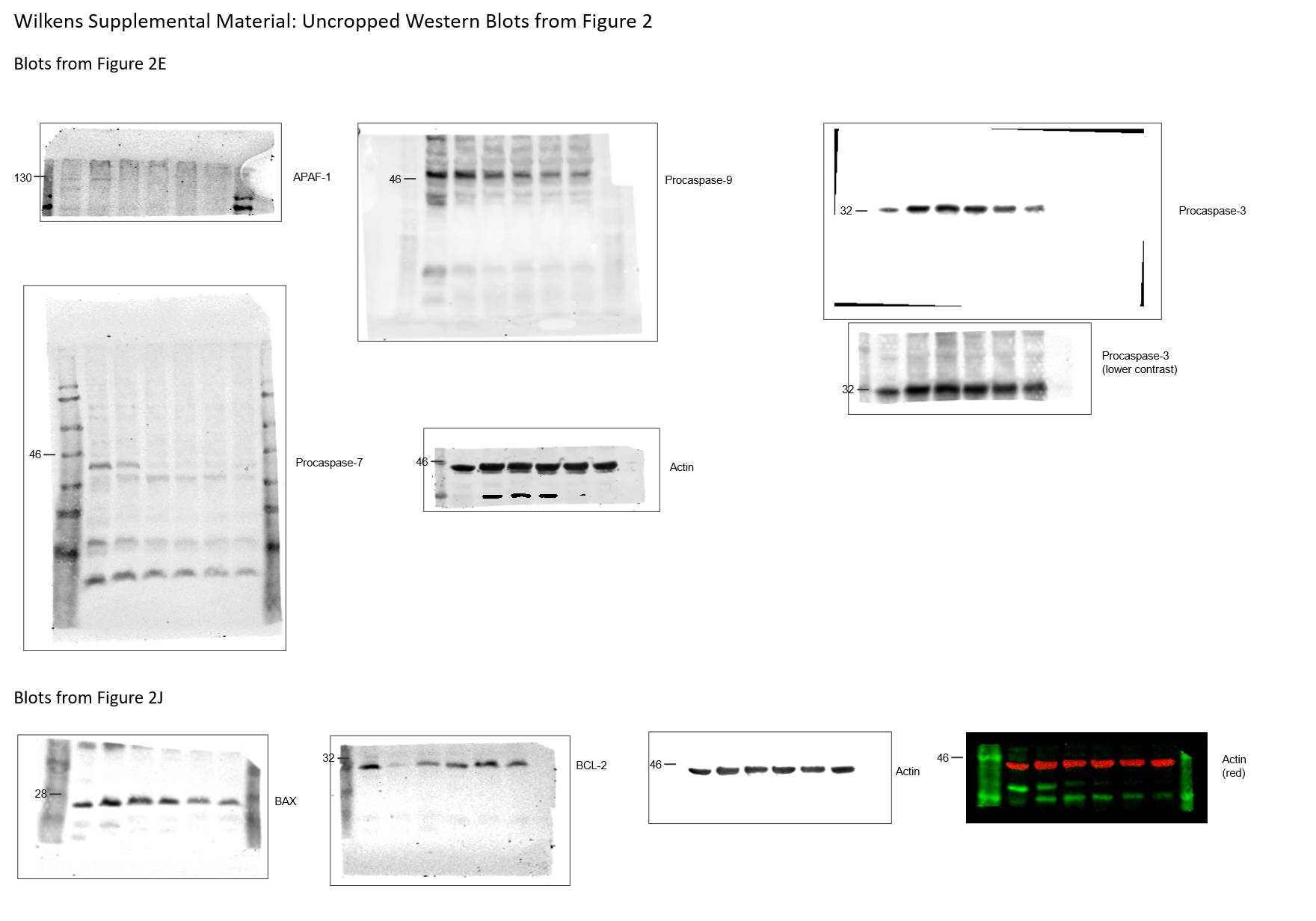

Supplement: Supplementary file 9 — Supplemental Material Uncropped Blots 1 [file 41419_2022_5340_MOESM9_ESM.tif]

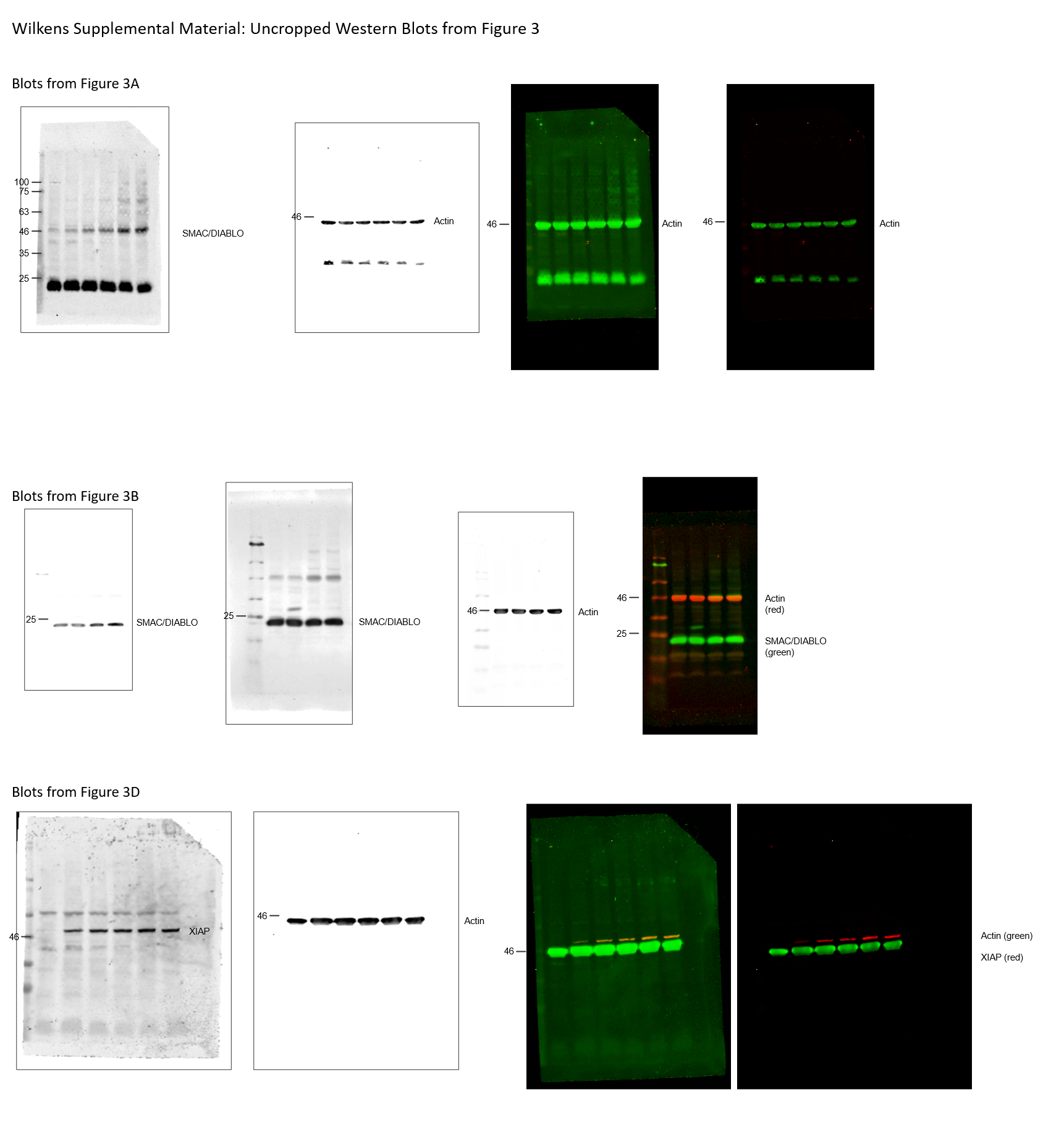

Supplement: Supplementary file 10 — Supplemental Material Uncropped Blots 2 [file 41419_2022_5340_MOESM10_ESM.tif]

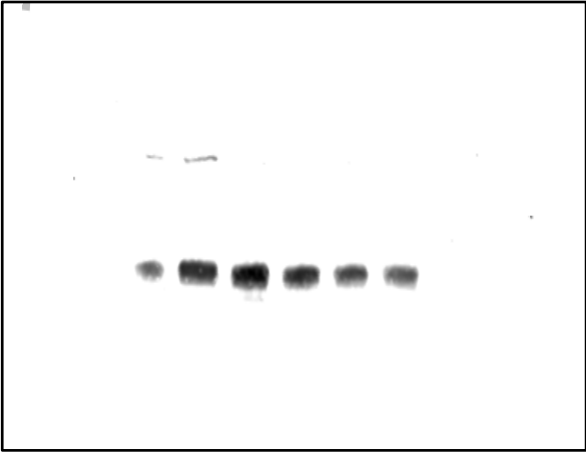

caspase3

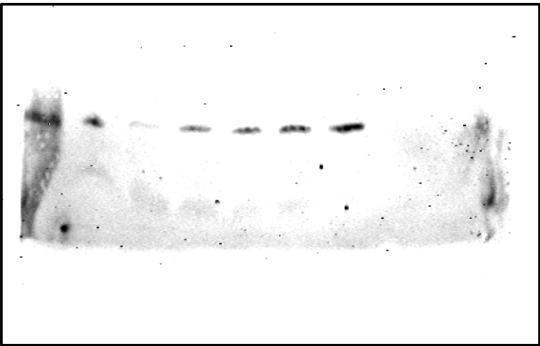

BCL2

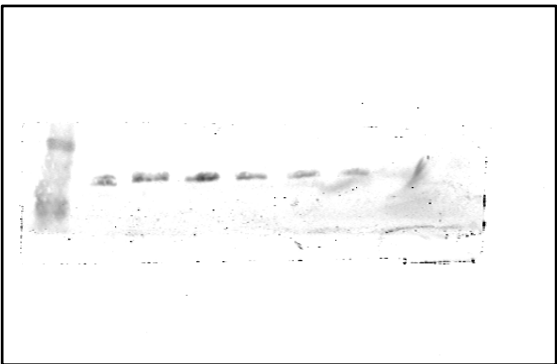

Bax

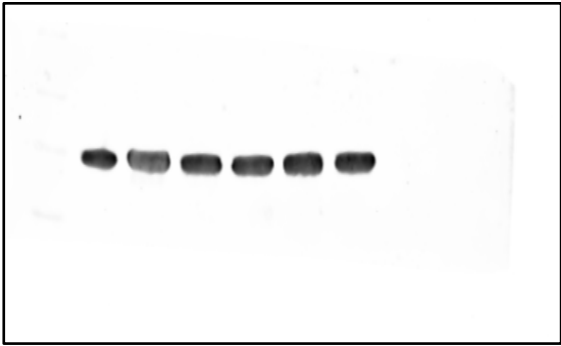

actin

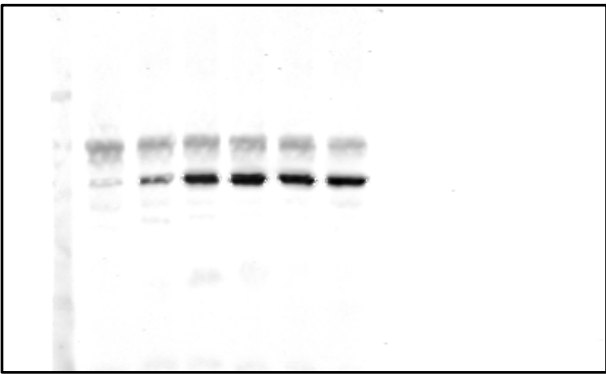

XIAP

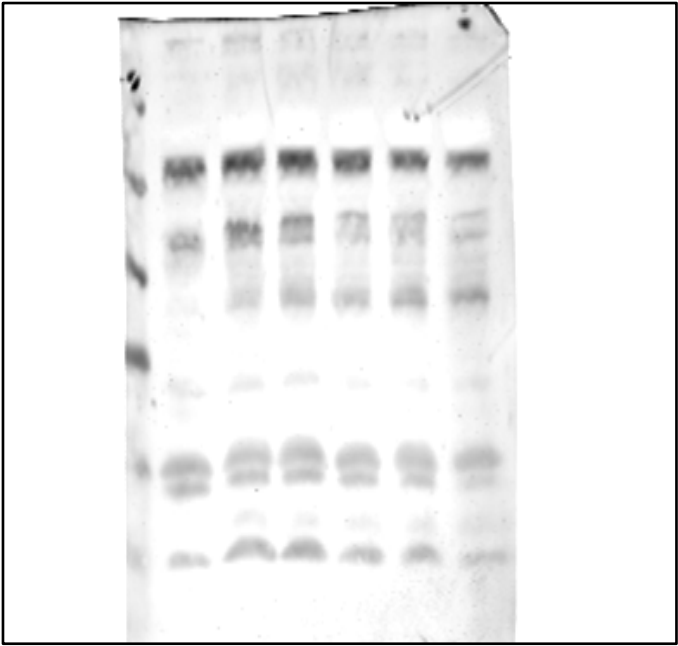

caspase9

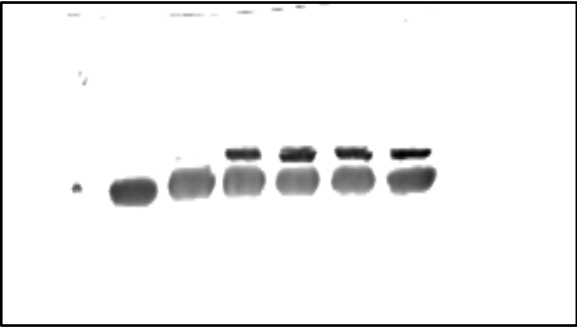

actin

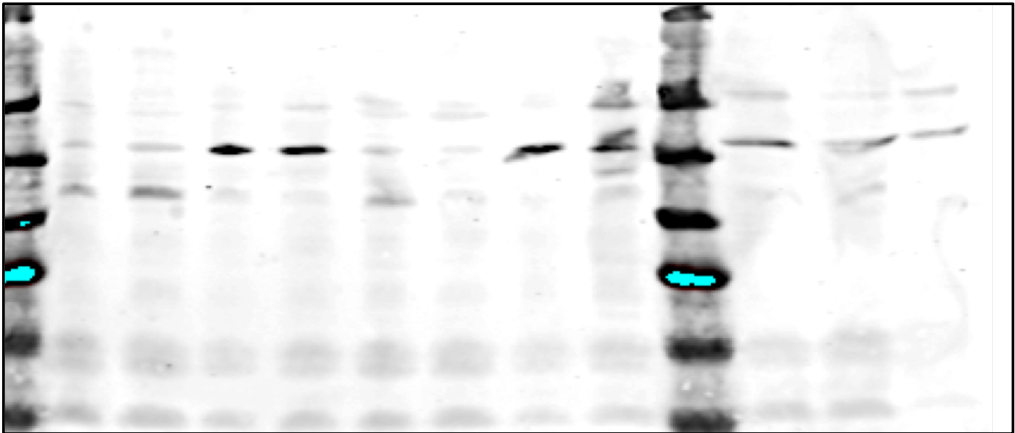

XIAP

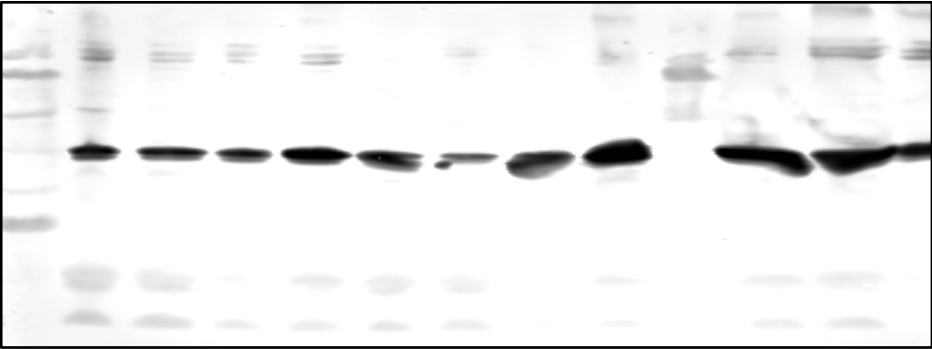

actin

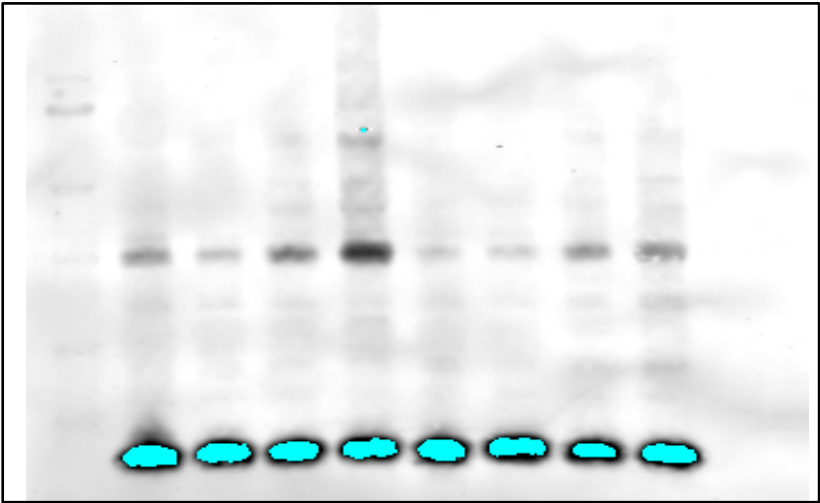

SMAC

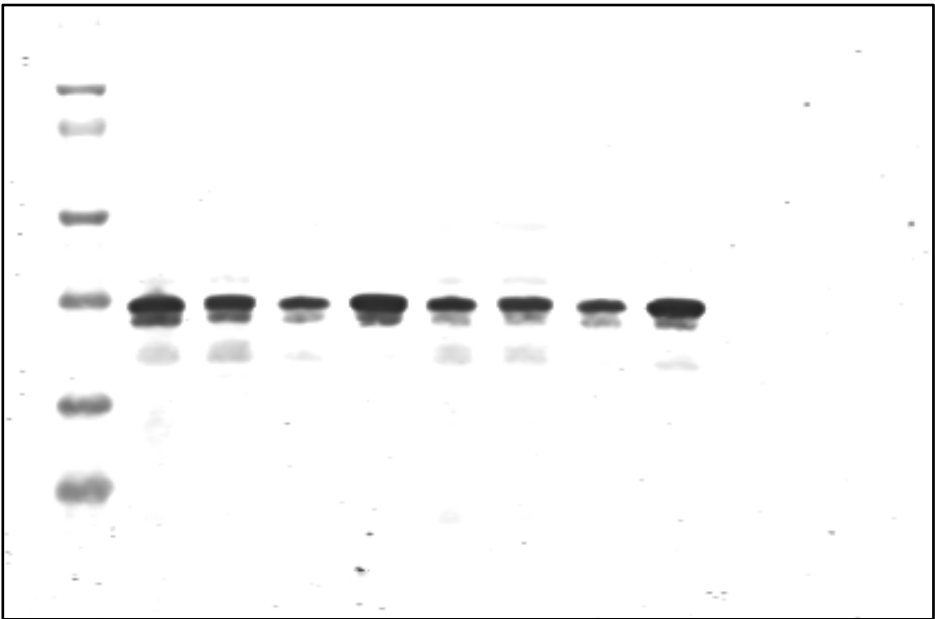

actin

Supplement: Supplementary file 11 — Supplemental Material uncropped blots 3 [file 41419_2022_5340_MOESM11_ESM.pdf]

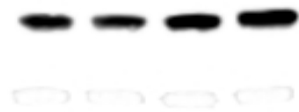

AKT

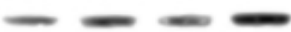

Phospho AKT

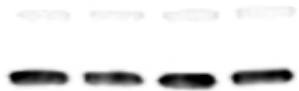

actin

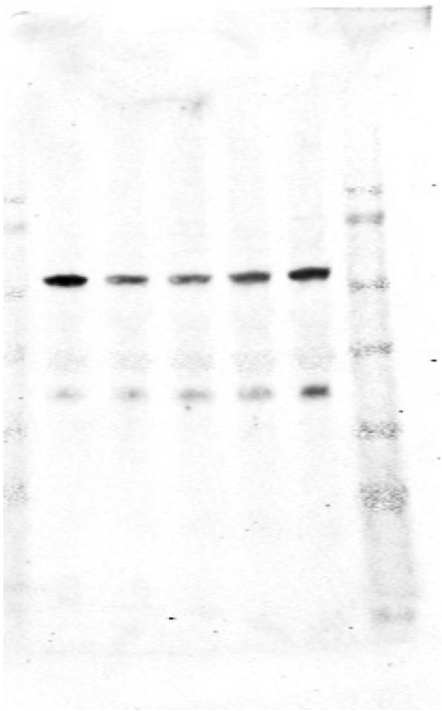

Phospho AKT

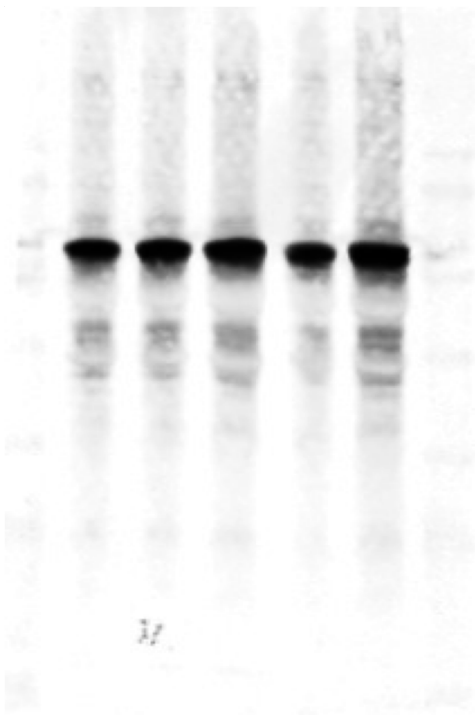

AKT

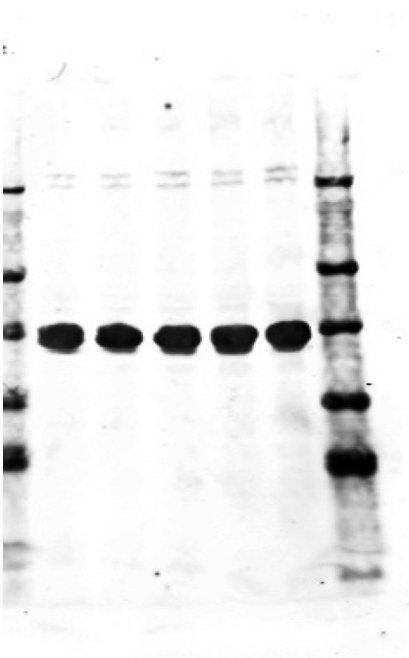

actin

Supplement: Supplementary file 12 — Supplemental Material uncropped blots 4 [file 41419_2022_5340_MOESM12_ESM.pdf]
